# Circle Method for Robust Estimation of Local Conduction Velocity High-Density Maps From Optical Mapping Data: Characterization of Radiofrequency Ablation Sites

**DOI:** 10.3389/fphys.2022.794761

**Published:** 2022-08-12

**Authors:** Jimena G. Siles-Paredes, Christopher J. Crowley, Flavio H. Fenton, Neal Bhatia, Shahriar Iravanian, Italo Sandoval, Stefan Pollnow, Olaf Dössel, João Salinet, Ilija Uzelac

**Affiliations:** ^1^ Graduate Program in Biotechnoscience, Federal University of ABC, São Paulo, Brazil; ^2^ HEartLab, Federal University of ABC, São Paulo, Brazil; ^3^ Georgia Institute of Technology, School of Physics, Atlanta, GA, United States; ^4^ Division of Cardiology, Section of Electrophysiology, Emory University Hospital, Atlanta, GA, United States; ^5^ Karlsruhe Institute of Technology (KIT)/Institute of Biomedical Engineering, Karlsruhe, Germany

**Keywords:** conduction velocity (CV), optical mapping, delayed activation, catheter ablation, local activation time, conduction slowing

## Abstract

Conduction velocity (CV) slowing is associated with atrial fibrillation (AF) and reentrant ventricular tachycardia (VT). Clinical electroanatomical mapping systems used to localize AF or VT sources as ablation targets remain limited by the number of measuring electrodes and signal processing methods to generate high-density local activation time (LAT) and CV maps of heterogeneous atrial or trabeculated ventricular endocardium. The morphology and amplitude of bipolar electrograms depend on the direction of propagating electrical wavefront, making identification of low-amplitude signal sources commonly associated with fibrotic area difficulty. In comparison, unipolar electrograms are not sensitive to wavefront direction, but measurements are susceptible to distal activity. This study proposes a method for local CV calculation from optical mapping measurements, termed the circle method (CM). The local CV is obtained as a weighted sum of CV values calculated along different chords spanning a circle of predefined radius centered at a CV measurement location. As a distinct maximum in LAT differences is along the chord normal to the propagating wavefront, the method is adaptive to the propagating wavefront direction changes, suitable for electrical conductivity characterization of heterogeneous myocardium. In numerical simulations, CM was validated characterizing modeled ablated areas as zones of distinct CV slowing. Experimentally, CM was used to characterize lesions created by radiofrequency ablation (RFA) on isolated hearts of rats, guinea pig, and explanted human hearts. To infer the depth of RFA-created lesions, excitation light bands of different penetration depths were used, and a beat-to-beat CV difference analysis was performed to identify CV alternans. Despite being limited to laboratory research, studies based on CM with optical mapping may lead to new translational insights into better-guided ablation therapies.

## 1 Introduction

Persistent cardiac arrhythmia can lead to heart failure, and heart failure can lead to arrhythmia. atrial fibrillation (AF) is the most common sustained cardiac arrhythmia, affecting 2.9% of the worldwide population (Benjamin et al., 2019). AF is associated with significant hemodynamic and thromboembolic complications (Zimerman et al., 2009). In comparison, ventricular tachycardia (VT) usually occurs in structurally diseased hearts.

Conduction velocity (CV) slowing is one of the determinants for AF vulnerability (Narayan et al., 2011), preceding AF initiation (Lalani et al., 2012), and is responsible for AF perpetuation (Shinagawa et al., 2000). VT initiation and persistence also depend on CV slowing (Moe et al., 1964; Allessie et al., 1977; Rensma et al., 1988). When an excitation wave encounters a zone of conduction block, it may propagate around the zone and reenter the previously unexcited region, reexciting it, repetitively (Rudy, 2012). As such, CV slowing facilitates reentry (Nattel et al., 2005) as for a reentrant wave to encounter an excitable tissue recovered from refractory phase, the propagating time around the block zone must be longer than the refractory period (Spector, 2013).

At the cellular level, CV slowing results from ionic remodeling or cell-to-cell uncoupling, leading to decreased excitability. For example, dilatation of the left atrium results in gap junctions remodeling (Takeuchi et al., 2006) and formation of atrial interstitial fibrosis, resulting in CV slowing due to reduced electrical conductivity between myocytes and fibroblasts (Vasquez et al., 2010; Thompson et al., 2011). Dilatation of ventricular tissue also reduces electrical conductivity, resulting in CV slowing (El-Sherif et al., 1987). This leads to dispersion of repolarization (Kuo et al., 1983), contributing to spatially discordant alternans (Chen et al., 2017; Uzelac et al., 2017, 2021), and arrhythmia susceptibility. Among these reasons, it is important to identify local CV changes at the high spatiotemporal resolution to identify and characterize regions of delayed activation to better understand the mechanism leading to arrhythmia (Irie et al., 2015b).

Radiofrequency catheter ablation (RFA) is the most effective invasive treatment for the termination and prevention of AF and VT recurrence, creating tissue lesions through thermal injury. Tissue sustains heating damage in direct contact with a catheter tip via resistive heating, while deeper tissue is damaged through convective heat transfer. Lesion formation is based on the assumption that heat transfer has a predictable profile in homogeneous tissue, and the profile of RFA-created lesions depends on many factors such as delivered RFA power and duration, temperature increase, catheter pressure force, catheter–tissue impedance, and the location of the ground patch.

While the lesion profile is predictable for homogeneous myocardium, persistent AF or VT may be caused by the reentrant waves originating from islets of heterogeneous myocardium within the scar, which can be buried inside the myocardial wall. The common RFA strategy is to identify clinically relevant scar tissue and deliver ablative energy to homogenize the scar. However, the effects of RFA on fibrotic tissue are poorly understood, as heat transfer prediction is challenging in contrast to heterogeneous tissue. Lesion formation depends on electric impedance and increases within scar tissue. Only 10% of RFA applications for scar homogenization resemble the expected lesion pattern (Barkagan et al., 2019). Additionally, adipose cells effectively shield the surrounding myocytes from RFA thermal injury (Sasaki et al., 2015). This imposes challenges in ablation treatment for VT, as one of the ablation goals is to create a lesion across the thick ventricular wall, which was significantly improved with the advent of irrigated catheter tips (Wittkampf and Nakagawa, 2006).

### 1.1 Measurement of CV

#### 1.1.1 Clinical Practice: Catheter Mapping-Based Measurement of CV

CV measurement has multiple potential benefits in clinical practice, especially during ablation procedures of complex arrhythmias. These benefits include mapping and localizing the substrate suitable for ablation and assessing the quality and depth of ablation lesions. However, CV measurement is not a standard part of such procedures despite all the potential benefits due to technical difficulties. Accurate determination of CV changes at a high spatiotemporal resolution to identify and localize areas of delayed activation is challenging in clinical practice. Instead, the focus during ablation is on the amplitude and fractionation of local electrograms. On the electrograms, the areas of interest for ablation commonly present as low-amplitude potentials and are characterized as arrhythmogenic substrates (Fukumoto et al., 2016; Kim et al., 2020). Anatomically, the areas represent islets of heterogeneous myocardium within the scar and can be alternatively identified in late gadolinium enhancement cardiac magnetic resonance imaging in atria (Fukumoto et al., 2016) or ventricles (Malaczynska-Rajpold et al., 2020).

Traditionally, substrate mapping before ablation is performed point by point with the help of a steerable mapping/ablation catheter under the guidance of a 3D electroanatomical mapping system. The success or failure of ablation to terminate persistent AF or VT depends on arrhythmia complexity, electrical catheter mapping type, and the sampling density. Bipolar electrograms are known to exhibit directional dependence on the propagating wavefront (Haines, 1993; Foppen, 2009). In contrast, unipolar electrograms may be contaminated with far-field electrical potential (Prystowsky, 2008; Allessie, 2014; Zaman et al., 2017), masking the myocardial activation time and misrepresenting the true electrophysiological state (Zaman et al., 2017). These technical challenges, along with uncertainties in electrode locations, hinder accurate CV measurement (Berenfeld et al., 2011; Zaman et al., 2017).

Fortunately, there have been recent advances in catheters and electrophysiology systems that render CV measurement in clinical settings feasible. These advances originated from the realization that CV measurement (both velocity and direction) is beneficial and, second, requires the relative geometry of recording electrodes to be tightly constrained. Under these conditions, the lesson learned from CV measurement of optical mapping data (on a grid) can be applied to clinical recordings. One such system is the Advisor HD grid catheter (Abbott Technologies, Minneapolis, MN), which forms a relatively rigid and flat 4 × 4 electrode array with known geometry and small electrodes (Hong et al., 2019; Hsen et al., 2020).

One emerging application of CV measurement in clinical electrophysiology is mapping VT substrates during sinus rhythm. For example, the isochronal late activation maps (ILAM) method is based on generating isochronal maps from local activation and identifying slow zones from isochronal crowding (Irie et al., 2015a; Aziz et al., 2019). It is expected that a more accurate CV measurement using grid catheters should improve the applicability of this technique.

#### 1.1.2 Basic Science: Optical Mapping-Based Measurement of CV

Conduction depends on fiber direction (anisotropy), tissue heterogeneity such as trabeculated or fibrotic tissue, aging, local ischemia, inflammation, or heart failure. Optical mapping directly measures transmembrane action potential (AP) at a high spatial resolution, achieving sub-millimeter mapping density. Therefore, it is a method of choice in basic science research to study cardiac electrophysiology from cells to tissue and whole heart level. As optical mapping can be used as a tool in studies of different end goals, published methods for CV estimation based on optical mapping differ (Tu et al., 1997; Mironov et al., 2008; Linnenbank et al., 2014; Doshi et al., 2015), with no one-fits-all method. While most CV methods are based on either polynomial surface fitting or a single vector approach, additional enhancements are often needed to tailor a method for the particular study aim.

In this study, we propose a robust method, termed the circle method (CM), for accurate estimation of local CV (both magnitude and direction) to evaluate the effects of RFA from optical mapping measurements. Ablated areas were localized, and spatial extent and depth of RFA-created lesions were characterized by examining the CV maps before and after ablation and identifying beat-to-beat CV alternans after the ablation.

## 2 Methods

### 2.1 Computer Simulations

Simulations were based on Fenton–Karma’s three variables model (Fenton and Karma, 1998) and were carried out using the explicit Euler method for the variables. The integration time step was *dt* = 0.05 *ms*, space discretization *dx* = 200 *μm*, and the standard diffusion coefficient of 0.001 *cm*/*ms* ^2^ was used. Tissue level simulations were performed in a 2D isotropic monodomain with 256 × 256 cells $\left(∼26\;cm^{2}\right)$ . The area is large enough to investigate the effect of CV reduction. The ablated region was modeled as the area of decreased excitability by increasing the time constant of the fast inward *Na* ^+^ current from 0.4 to 0.55 *ms*. Low excitability is associated with a larger threshold stimulus needed to support AP propagation and is associated with CV slowing. With the model parameter change, modeled RFA area was not excitable. Inside the ablated area, wavefront propagation was only possible due to electronic coupling, significantly decreasing CV.

To test the proposed CM method in predicting RFA size and location as a function of measurement noise, white Gaussian noise was added to the numerical data with different signal-to-noise ratios (SNRs): 5, 10, 15, 20, 25, 30, and 60 dB (the amplitude ratio, respectively: 1.78, 3.16, 5.62, 10, 17.78, 31.62, and 1,000). Then, LAT, CV maps, and estimated ablation area (size and location) are obtained to evaluate the effect of different noise levels. The surrogate CV maps, obtained with different noise levels, were compared with the reference CV map with no added noise, using the 2D Pearson’s correlation coefficient. The 2D Pearson’s correlation coefficient was calculated by choosing two different segments of the domain, a segment mainly containing the ablated area and a segment containing almost the entire simulation domain, with the ablated area at the center. The Dice similarity coefficient (Dice, 1945) was used to evaluate the impact of noise by comparing cropped CV maps with the reference CV map to test for noise effects on the accuracy in the determination of the ablated area location.

### 2.2 Heart Excision and Experimental Setup

Optical mapping imaging with transmembrane voltage (V_*m*_) sensitive dyes was used to characterize RFA-created lesions, analyzing high-density CV maps before and after RFA in *ex-vivo* superfused isolated rat atria (*N* = 2), arterially perfused guinea pig heart (*N* = 1), and explanted human hearts (*N* = 2). Motion was suppressed for guinea pig and human hearts with (-)-Blebbistatin (Cayman Chemicals) at 1.8 *μ*M concentration in Tyrode perfusate. All RFA procedures were performed using a high-frequency desiccator (Bowie).

**FIGURE 1:**
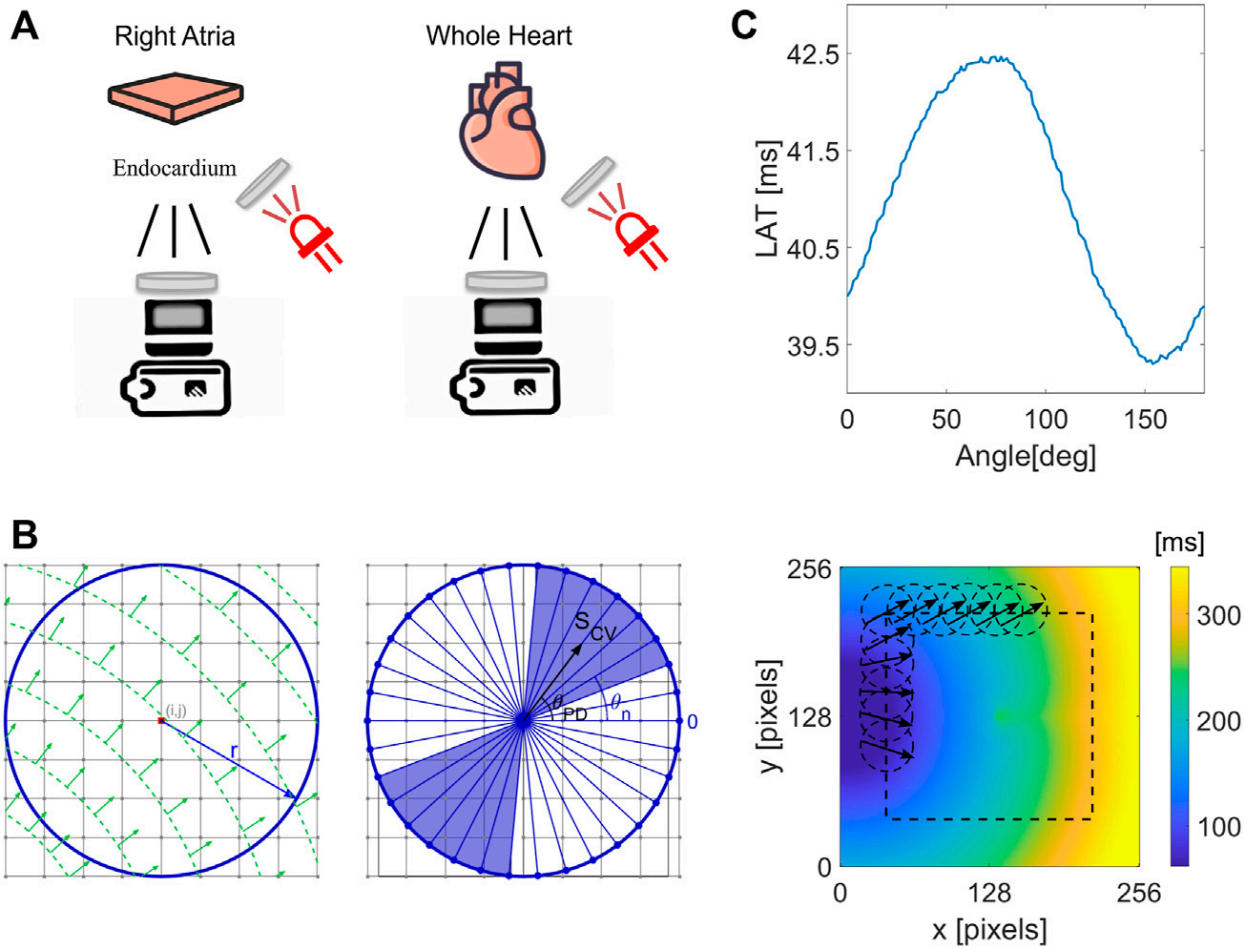
Illustration of the experimental setup and the CM method application to obtain local CV and CV maps. **(A)** For isolated rat atria, RFA was performed in steps of increasing ablation duration of 0.5, 1, 1.5, 2, 2.5, 3, and 4 s, with 2 min pause between the steps. The ablation was performed on the epicardial side with optical mapping measurement on the endocardial side. The ablation was performed on the ventricular tissue for the whole guinea pig heart and human heart preparations. Collimated and bandpass-filtered LED light was used to excite the *V*_*m*_ dye. Emanating fluorescent light was passed through the long-pass filter in front of the EMCCD camera sensor. **(B)** Illustration of the circle method, evaluating LATs differences at the points along a circle of radius r, centered at a grid point (i,j). Left, example of a wave propagating at 45°. Right, a set of test CV values, *S* (*θ*_n_), which is evaluated by taking the difference in LATs along diameters (blue lines) at different angles *θ*_*n*_. The circle method evaluates the resulting conduction velocity by performing a geometrically weighted average of *S* (*θ*_n_) over an angular range around *θ*_PD_ (blue shaded region). **(C)** Top, an example of a set of LAT differences for different chords, with a clear maximum along the chord normal to the propagating wavefront along vector **S_CV_** as in **(B)**. Bottom, application of the CM method at every pixel in the LAT map inside the dashed rectangular region, obtaining local CV directions.

Heart excision and experimental setup for isolated rat atria have been described in detail elsewhere (Pollnow, 2018). In brief, the right atria from two Fisher rats were dissected by cutting along the tricuspid valve to the superior vena cava, fixated in a bath, superfused with Krebs–Henseleit solution, and stained with Di-4-ANEPPS V_*m*_ sensitive fluorescent dye. The dye was excited using a green LED of 525 nm center wavelength (Cairn Research). Emitted V_*m*_ fluorescence from endocardial tissue was passed through the 635DF55 filter (Omega Optical) on the camera side. The sequence of images was acquired using an EMCCD camera (Evolve Delta 512, Photometrics) at a sampling frequency of 868 Hz with a binning factor of 2 and a resolution of 82 × 82 pixels, equivalent to a spatial resolution of 128 × 128 μm/pixel corresponding to a 10.5 × 10.5 mm field of view (Figure 1A). The atrial epicardium was stimulated (6.7 Hz) with a unipolar electrode. The RFA was performed approximately at the center of the epicardium as a sequence of RFA steps, increasing RFA time duration in each step while keeping the RFA power constant at 10 W. The ablation procedure was performed using a tungsten electrode of 0.4 mm diameter (Pollnow, 2018).

Heart excision and experimental setup in Langendorff-based whole heart perfusion using whole isolated guinea pig heart have been described in detail elsewhere (Uzelac et al., 2021). The RFA procedure was performed on the epicardial side of the left ventricle, using a 0.4-mm-wide tungsten electrode for 20 s with power set to 10 W (Figure 1A).

The cardiac surgeon team performed human heart excisions during the heart transplantation procedure. Immediately after receiving the heart from the surgical team, ice-cold cardioplegia solution was flushed through the right and left coronary arteries to protect the myocardium against ischemia during transportation to our optical mapping lab within 20 min while maintaining arrested heart temperature around 4°*C*. The left and right coronaries were separately perfused in the whole heart preparation for epicardial optical mapping. The human left ventricular wedge preparation was used for optical mapping of the endocardium. The marginal artery was cannulated, and the cannula was secured with a surgical silk ligature. Leaks in the wedge preparation were secured by clamping the wedge preparation around the cut regions. The whole heart and the wedge preparations were placed in an oval heated chamber maintained at 37°*C* while continuously perfused with oxygenated Tyrode solution (Ng et al., 2014) also kept at 37°C and oxygenated with the mixture of 95% O_2_/5% CO_2_. Coronary pressure of 70 mmHg was kept constant during the experiments. RFA was performed with power set to 40 W, using a non-irrigated electrode with a blunt tip.

Guinea pig and human hearts were stained with V_*m*_ sensitive dye Di-4-ANBDQPQ (JPW-6003) (Potentiometric dyes), with 0.25 mg of the dye for whole guinea pig heart, and 0.5 mg of the dye for each human ventricle. The dye was prepared as a stock solution previously dissolved in pure ethanol at a 1 mg/ml ratio. Two red LEDs with the center wavelength at 660 nm were used as light excitation sources for the V_*m*_ dye (Figure 1A). The LED light was collimated with a plano-convex lens (ThorLabs) and bandpass filtered with a 660/10 mm filter (Edmund Optics). Additionally, two green LEDs with a center wavelength of 525 nm were used for the optical mapping measurements performed on the human heart endocardium. The LED light was collimated with a plano-convex lens (ThorLabs) and bandpass filtered with a 520/10 mm filter (Edmund Optics). A custom-designed two-channel LED driver was used, with the ability to switch the excitation light bands in sync with the camera frame rate (donated from Aleksa Tech). The emitted fluorescence was passed through a 700 nm long-pass filter (Chroma) on the camera side. The sequence of images was acquired at 500 Hz using an EMCCD camera (Evolve 128, Photometrics) at a resolution of 128 × 128 pixels.

In the post-processing, baseline drift for each pixel trace was removed by applying a low-pass Kaiser Window FIR filter with a stop-band frequency of 1 Hz and a pass-band frequency of 0.5 Hz. The output signal from the FIR filter, representing the baseline drift, was subtracted from the raw pixel trace. The difference was divided with the baseline drift signal, obtaining the relative change in fluorescence ΔF/F (Uzelac et al., 2019). To boost SNR, approximately 40 optical APs (OAPs) were stacked (ensemble averaged) with a period equaling two beats upon reaching steady-state conditions, significantly reducing the noise (Uzelac and Fenton, 2015). In the next step, the traces were temporally filtered using an anisotropic 1D diffusion filter (Perona and Malik, 1990; Gerig et al., 1992), which preserves the OAP upstroke.

LATs were obtained by linear fit along the OAP upstroke and determining the 50% rise of the OAP upstroke. Contrary to bipolar electrograms, where activation time determination is based on the maximum of the first derivative, OAP signals are inherently spatially averaged across clusters of cells corresponding to a single pixel trace. As the sampling rate is generally lower, the first derivative method is less accurate than the 50% approach (Efimov et al., 2004; Fedorov et al., 2009; Walton et al., 2012; Bastos-Filho, 2021). Obtained LAT maps were subsequently minimally filtered using an anisotropic 2D diffusion filter, which preserves sharp boundaries in LAT maps (Perona and Malik, 1990; Gerig et al., 1992).

### 2.3 Circle Method (CM)

By assuming a planar wavefront propagation inside a circle of radius *r* (Figure 1B), centered around each LAT grid point, the CV can easily be determined. This can be done by first calculating differences in the LAT across the endpoints of chords passing through the center of the circle. From the LAT differences, an effective conduction speed, *S*(*θ*), can be calculated as a function of the chord’s orientation angle, *θ*. The maximum of *S*(*θ*) corresponds to the true conduction speed along the propagation direction, *S*(*θ*_*PD*_) (Figure 1C). The evaluation of *S*(*θ*_*PD*_) incorporates measurements along a single path, centered at the LAT grid point. To reduce the effect of LAT measurement uncertainty, CV along neighboring chords can be incorporated.

To reduce noise in the determination of the CV, conduction speeds along chords lying within the range, $\left(\theta_{\text{PD}}-\frac{1}{2}\Delta \theta ,\theta_{\text{PD}}+\frac{1}{2}\Delta \theta \right)$ , are combined to calculate a CV that is spatially averaged over the hourglass-shaped area subtended by these chords (Figure 1B right). Because chords within this range do not all lie along the direction of propagation, the conduction speeds are enhanced to account for this geometry before combining. To account for the misalignment, $\vec{S}\left(\theta \right)$ is back projected onto $\vec{S}\left(\theta_{PD}\right)/\|\vec{S}\left(\theta_{PD}\right)\|$ . The resulting conduction speed can be calculated from the average of these back-projected speeds as,

$$S_{\text{CV}}=\frac{1}{N}\overset{N}{\underset{n=1}{\sum }}\frac{S\left(\theta_{n}\right)}{\cos\left(\theta_{PD}-\theta_{n}\right)},$$ (1)

for all *N* chords lying within the range spanned by Δ*θ*. For a detailed discussion of this equation, see Supplementary Material.

This method is equivalent to central differences with a separation of 2*r* grid points for waves propagating along the optical mapping grid; however, unlike central differences, the fidelity of the calculation does not depend on the propagating wavefront orientation. Additionally, interpolation is performed for LAT differences calculated along chords with endpoints that do not fall on LAT grid points. Incorporating conduction speed information along with many chords reduces uncertainty in the estimation of local CV.

For comparison with other methods, the CV maps from the numerical simulation, isolated rat atria, guinea pig heart, and whole explanted human heart, obtained with the CM and the finite-difference approach (FiD) (van Schie et al., 2021), were used. The distance between neighbors using the FiD method was set to 20 pixels, corresponding to *r* = 10 used for CM (diameter = 20 pixels). A MATLAB function for implementing the CM for a given LAT map is provided on GitHub at https://github.com/uzelaci/Circle_Method and https://github.com/HEartLab-ufabc/Circle_Method.

## 3 Results

### 3.1 Computer Simulations

**FIGURE 2:**
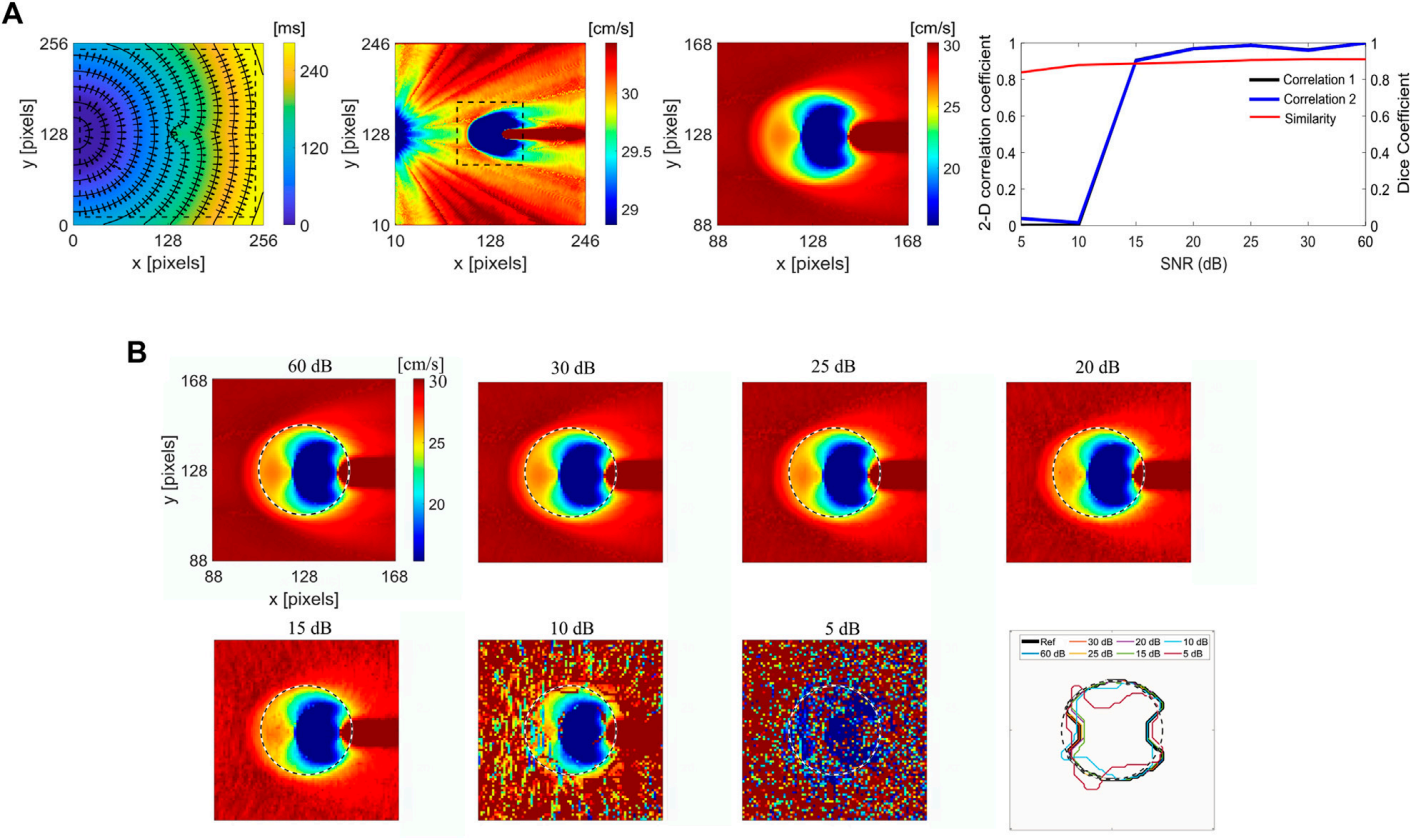
Numerical simulations of the RFA and effects of added noise on CV for the spatial determination of the ablation area. **(A)** Left, the LAT map of a simulated curved wavefront propagation with isochrone lines spaced 20 ms apart, and superimposed CV vector map showing CV magnitude and direction. The ablated area is modeled as a circular region, 20 pixels radius at the center. Middle, the corresponding CV maps from the LAT map for the whole domain and sub-domain (80 × 80 pixels) region around the ablated area outlined with an inscribed dashed rectangle. Right, 2D Pearson’s correlation coefficient for different SNR levels ranging from 5 to 60 dB, for the CV maps of different sizes on the left, 236 × 236 (Correlation 1) and 80 × 80 pixels (Correlation 2). The Dice similarity coefficient for the selected 80 × 80 pixels CV map around the ablated area. **(B)** Zoomed-in CV surrogate maps for different SNRs of 60, 30, 25, 20, 15, 10, and 5 dB, with the delineated boundary of the ablated area. The color bar range in all figures is set from the 5th to 95th percentile.

Figure 2A shows the LAT map of simulated curved wavefront propagation with isochrone lines and superimposed CV vectors, and corresponding CV maps for the entire domain and the selected area with dash line, around the ablated region for improved visibility. The ablated area was modeled as decreased excitability for a circular region, 20 pixels in radius, centered in the domain. Due to decreased excitability in the ablated area, propagation is only due to electrotonic coupling between the excitable and non-excitable (ablated) regions, resulting in a wave block for a large enough ablated area. A point stimulation was used to generate a curved propagating wavefront to capture wavefront effects. A 2D Person correlation (Figure 2A upper right) was used to compare the reference noise-free CV map and its surrogates (Figure 2B), obtained with different noise levels. Correlation 1 plot refers to the correlation coefficients obtained regarding the referent noise-free CV map of whole domain (236 × 236 pixels), and Correlation 2 for the subdomain around the ablated area (80 × 80 pixels). Dice similarity coefficient is calculated for the subdomain to compare the noise-free reference CV map and its surrogate maps with added noise.

Figure 2B shows the noise effect on the CV maps and ablation area segmentation under different SNR levels of 60, 30, 25, 20, 15, 10, and 5 dB. The correlation coefficients are similar for the SNR range between 60 and 15 dB, varying from 1 to 0.9. Correlation decreases for SNR < 15 dB, with no difference between the two correlation curves, and a significant difference for SNR < 10 dB (0.020). Dice similarity coefficient is less dependent on noise. For SNR = 5 dB, the coefficient is equal to 0.83, and increases to 0.91 for SNR = 60 dB. Even under high levels of noise (i.e., SNR < 5 dB), the ablated area could be segmented (i.e., size and location), showing a high level of similarity between noise corrupted CV maps and the reference noise-free CV map.

### 3.2 Effects of Ablation on CV: Isolated Rat Atria

**FIGURE 3:**
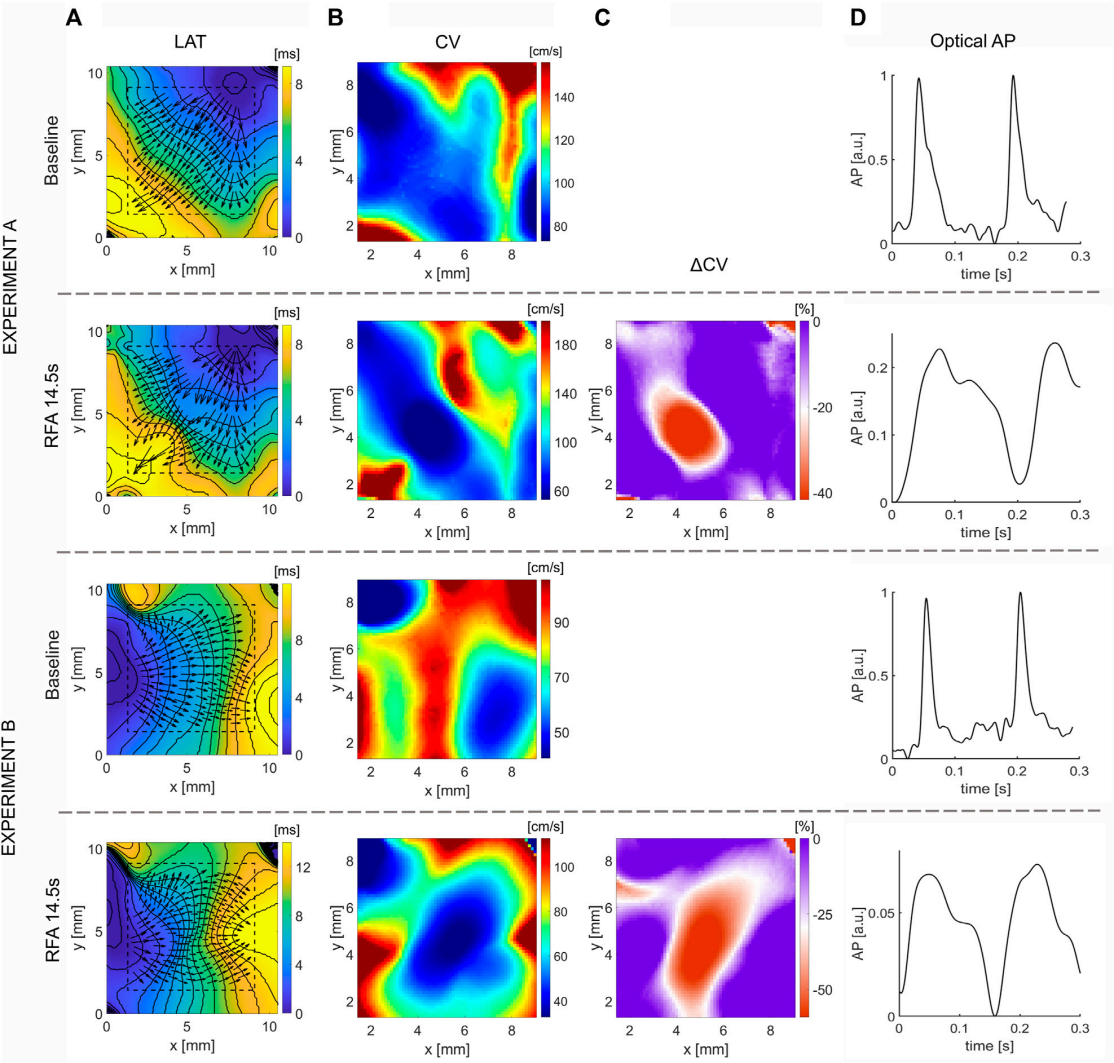
Effect of ablation on CV slowing on isolated rat atria. **(A)** LAT map obtained before (Baseline) and after RFA for the total duration of 14.5 s and power of 10 W, approximately at the center of the map, along with the superimposed CV vector map. **(B)** Respective CV maps obtained with the CM method for selected dashed square regions presented in **(A)** outline the ablated areas as regions of CV reduction. **(C)** Relative change, ΔCV, between the CV maps after and before the ablation, shows a large relative decrease of CV in the ablated areas, delineating the ablated area from non-ablated, suitable for characterization of the ablated area boundary. **(D)** Optical AP traces from a pixel inside the ablated area, normalized before ablation, show the reduction in AP amplitude and morphology changes. The color bar range in all figures is set from the 5th to 95th percentile, except for ΔCV maps’ upper boundary that was set to zero. Radius of 10 pixels (1.3 mm), in the CM, was used for CV calculation.

Figure 3A shows LAT maps obtained before and after 14.5 s of ablation for two experiments on isolated rat atria. In the experiment A, the wavefront propagates from the upper-right corner, and in the experiment *B* from the left side. The respective CV maps (Figure 3B) are calculated for the area outlined with a dashed line. A region of distinctly lower CV values depicts the location and shape of the ablated tissue, represented by a deep-blue shaded area. The relative difference in CV maps after and before the ablation for both experiments is shown in Figure 3C. A well-defined area where CV decreases is observable, delineating the region where ablation was performed. In both experiments, the ablated area resembles an elliptical shape of approximately 24 × 48 pixels for experiment A (2 × 4 mm) and 24 × 72 pixels (2 × 6 mm) for the experiment B. The OAPs inside these areas are presented in Figure 3D, depicting a higher amplitude reduction and change in morphological pattern.

### 3.3 Effects of Ablation on CV: Guinea Pig Ventricle

**FIGURE 4:**
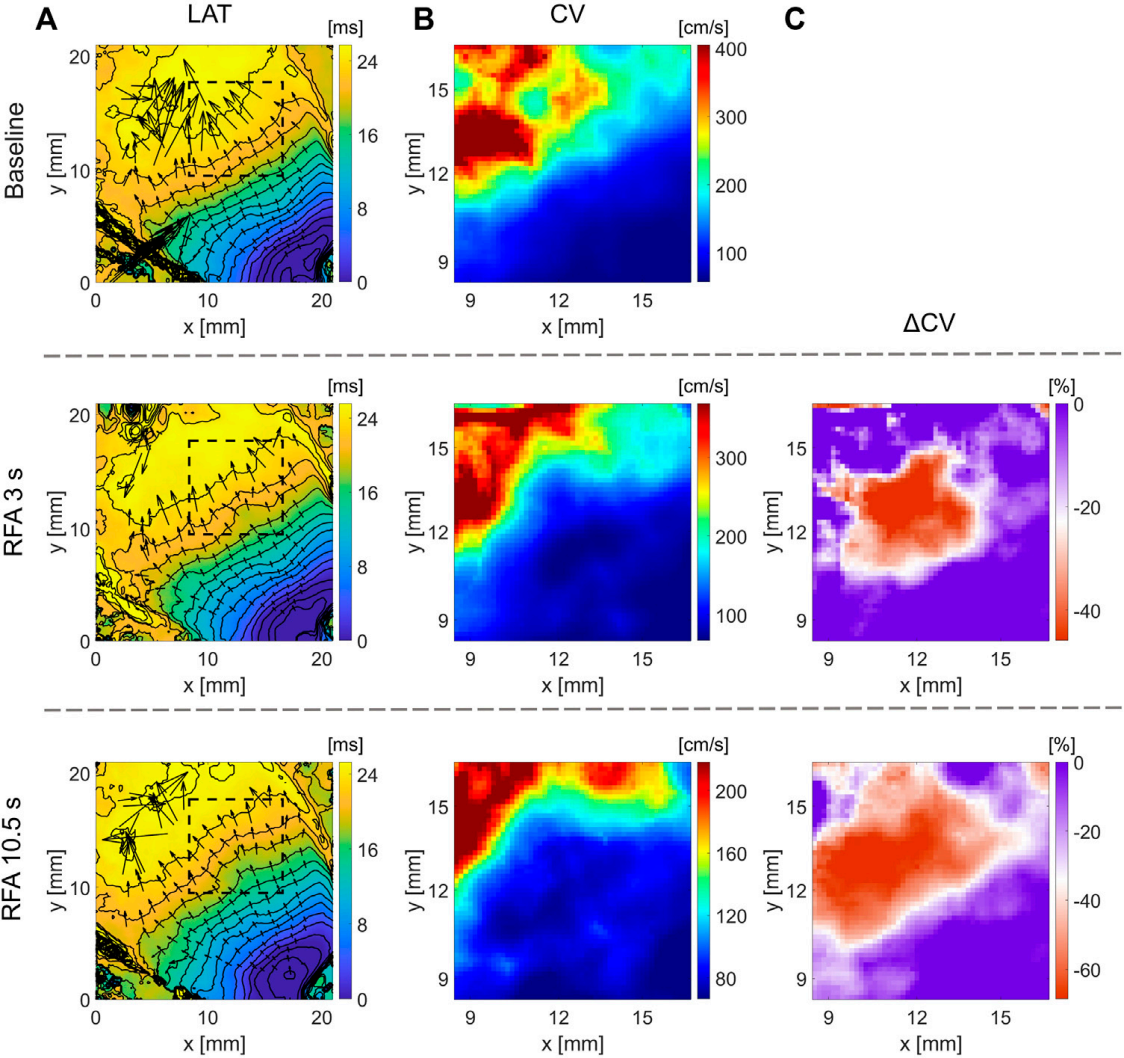
Effect of ablation on CV slowing on guinea pig ventricle for different RFA durations. **(A)** LAT maps obtained at baseline (top) and RFA duration of 3 s (middle) and 10.5 s (bottom), respectively. **(B)** Respective CV maps inside the dashed square regions, where ablation is performed, show CV decrease, with a larger CV decrease after longer RFA. **(C)** Relative difference in CV after and before ablation, ΔCV maps, outlines the ablated region as a distinct decrease of CV. The color bar range in all figures is set from the 5th to 95th percentile, except for ΔCV maps’ upper boundary that was set to zero. Radius of 10 pixels (1.6 mm), in the CM, was used for CV calculation.

Wavefront propagation across ventricular tissue is more complex than atrial tissue due to tissue heterogeneity and transmural wave propagation. Figure 4A shows the LAT maps obtained at baseline and after application of RFA (initially applied for 3 s and subsequently for 10.5 s). Figure 4B shows the respective CV maps, calculated from areas around the ablated region, within the dashed square region from Figure 4A. From the figure, it can be inferred that the area representing lower CV values expands in size, with an additional ablation of 10.5 s. Due to the inherent spatial variation in CV, unlike with CV maps of more homogenous rat atria, direct comparison of CV maps is more difficult. A CV map of relative change after and before the ablation, ΔCV map, was calculated for the two ablation steps shown in Figure 4C, showing the relative decrease in CV post-ablation. The maps delineate the elliptically shaped red-color-shaded ablated region.

### 3.4 Effects of Ablation on CV: Human Heart Ventricles

**FIGURE 5:**
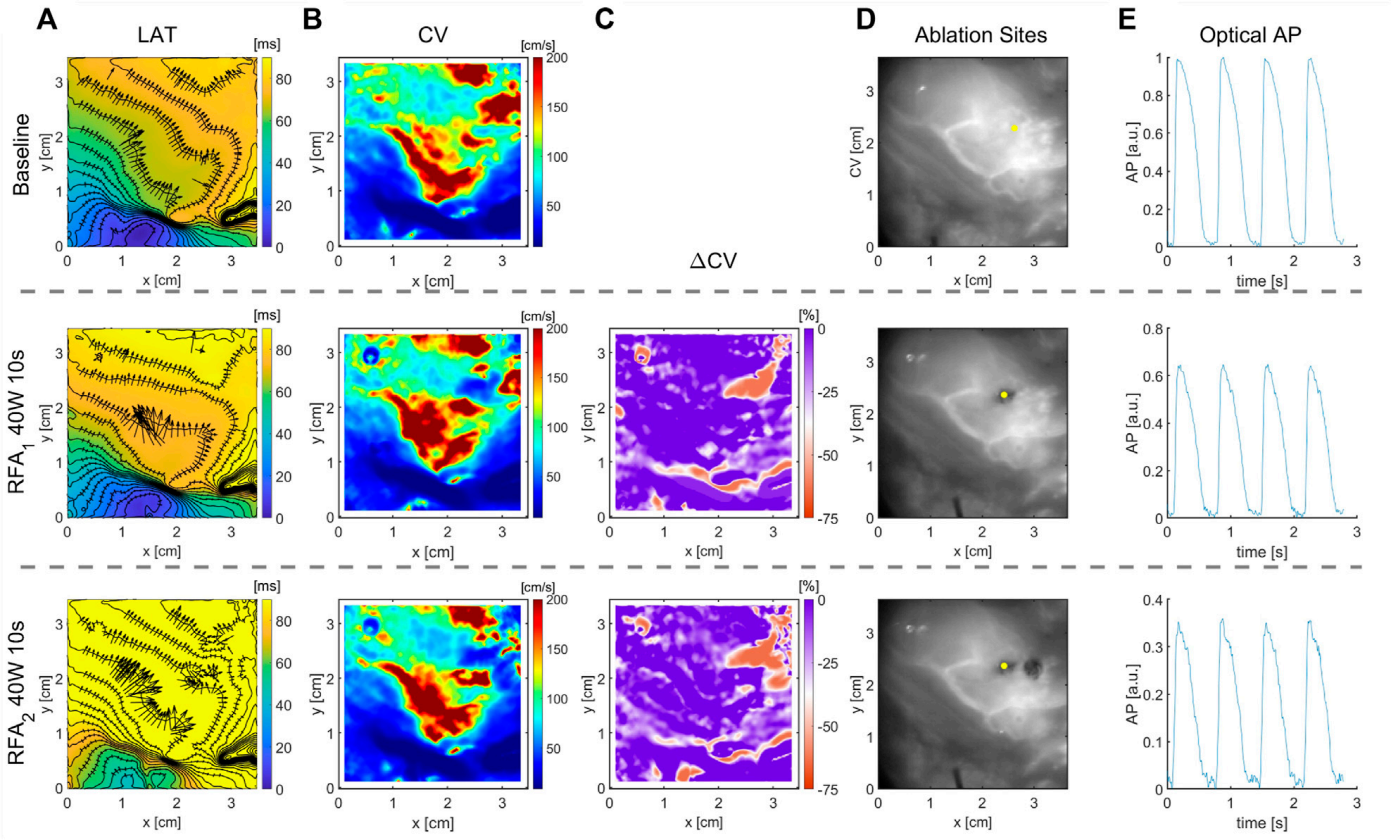
Optical mapping on whole explanted human heart for quantification of RFA-created lesion. RFA was performed on ventricle’s epicardial side in two steps, termed RFA_1_ and RFA_2_, with power set to 40 W for 10 s in each step. **(A)** LAT time maps show wavefront propagation slowing in the upper right region where the ablation was performed. **(B)** CV maps before and after ablation show characteristic CV slowing in the ablated region. **(C)** Change in CV obtained as the relative difference after and before ablation clearly outlines the red area where ablation was performed of a distinct CV decrease. **(D)** Black and white images captured using the optical mapping camera illustrate ablated regions. **(E)** Optical AP traces from a pixel marked with a yellow dot in **D** normalized before ablation. The traces show the relative change in fluorescence corresponding to transmembrane voltage change. The color bar range in all figures is set from the 5th to 95th percentile, except for ΔCV maps’ upper boundary that was set to zero. Radius of 4 pixels (1.1 mm), in the CM, was used for CV calculation.

**FIGURE 6:**
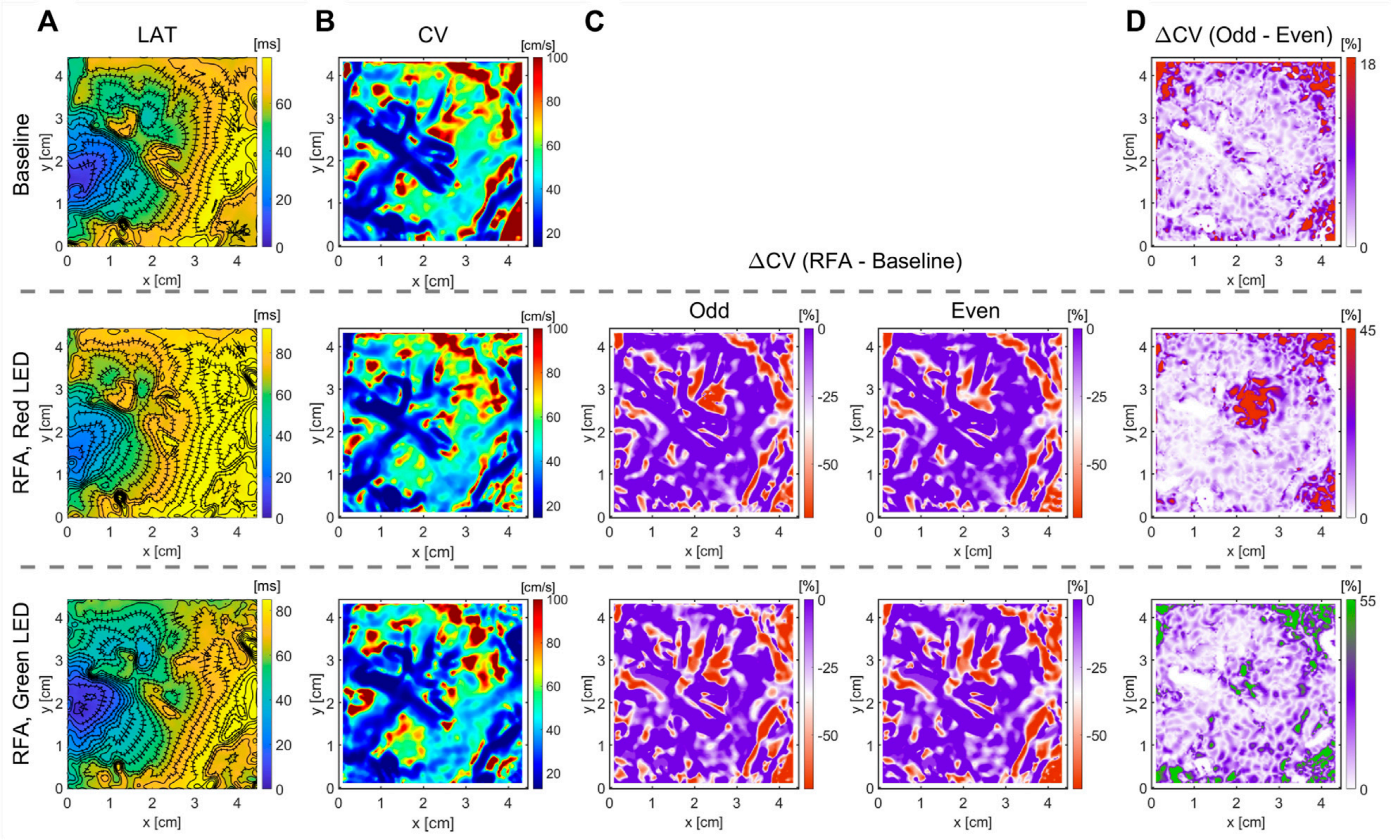
Optical mapping on the LV’s endocardium in wedge preparation for quantification of RFA-created lesion. The RFA was performed on a highly trabeculated endocardium for 20 s, and power set to 40 W. Different light excitation bands were used, a green LED of 525 nm center wavelength and a red LED of 660 nm center wavelength, to measure optical APs emanating from the endocardial surface and deeper transmural layers. **(A)** Due to the highly heterogeneous endocardium (Supplementary Figure S2), identification of the ablated area from LAT maps is non-trivial. **(B,C)** CV maps and the relative CV difference after and before the ablation do not clearly outline the ablated area, lacking characteristic CV slowing. **(D)** Relative difference in CV maps between subsequent beats (wavefront propagation), termed even and odd, elicits the CV differences due to AP amplitude alternans in the ablated area. Green LED light illumination of penetration depth limited to the endocardial surface layer, and the absence of alternans indicates a complete ablation of the surface layer. Illumination with a deep-red light band of deeper penetration depth enables measuring APs from deeper ventricular wall layers. The presence of CV alternans indicates the non-homogeneous or incomplete ablation across the ventricular wall (Supplementary Figure S2). The color bar range in all figures was set from the 5th to 95th percentile, except for ΔCV maps’ upper boundary that was set to zero. Radius of 3 pixels (1.05 mm), in the CM, was used for CV calculation.

Figures 5, 6 show optical mapping measurements on the whole explanted human heart with epicardial ablation and left ventricle in wedge preparation with endocardial ablation, respectively, using a blunt ablation electrode (Bowie). Hearts were explanted from patients undergoing heart transplantation, suffering from progressive heart failure and recurrent VT as a result of viral myocarditis. As such, ventricular tissue is highly heterogeneous, and the CV maps illustrate challenges in quantifying CV maps. Despite optical mapping achieving high density in comparison to contemporary electroanatomical mapping systems, LAT and CV maps illustrate the complex electrophysiology of the human ventricles. This observation is expected as the human endocardium can be highly trabeculated, and the presence of papillary muscle may hinder the mechanism of CV slowing (Supplemental Figure S2). For both hearts, epicardial and endocardial LAT and CV maps fail to delineate the ablated region. Moreover, for endocardial ablation (Figure 6), even the maps of relative CV difference before and after ablation are not much practical to characterize RFA lesion. Therefore, we modified our optical mapping protocol to overcome these challenges and performed optical mapping with light bands of different penetration depths to study beat-to-beat CV alternans in post-ablation. Analysis of relative CV maps difference for subsequent beats with green light band illumination does not show the presence of CV alternans, indicating a complete surface ablation. However, the same analysis with deeper red-light band illumination of greater tissue penetration depth shows CV alternans due to incomplete or non-homogeneous ablation across the ventricular wall. This observation agrees with the images of the RFA lesion taken after the experiment, showing the spatial extent of the lesion and its depth across the ventricular wall (Supplemental Figure S2).

## 4 Discussion

**FIGURE 7:**
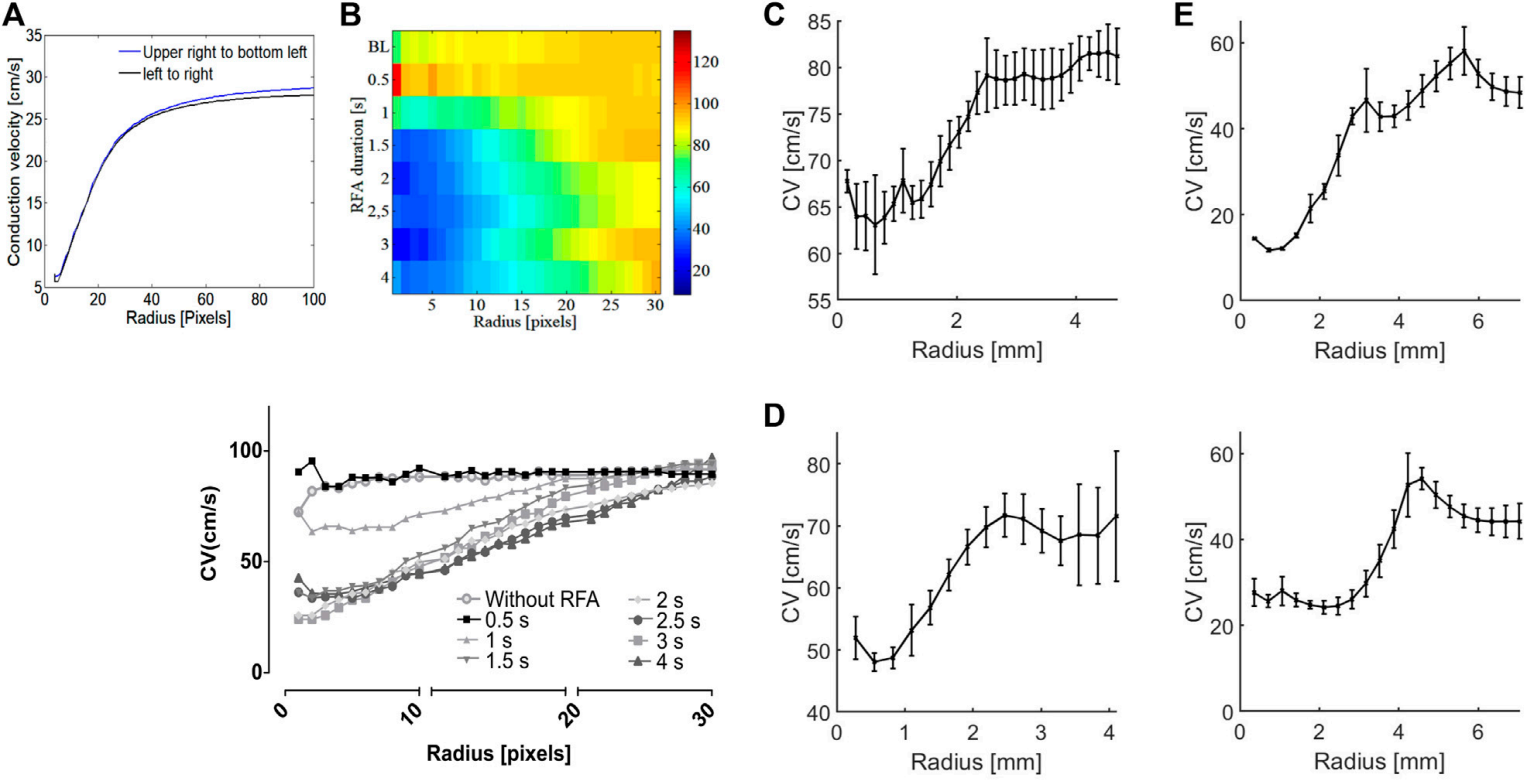
Estimation of the ablated area size based on the CM radius. **(A)** In numerical simulations, CV linearly increases with CM radius inside the ablated area of 20 pixels in radius, reaching a limit value outside the ablated area. The ablated area radius can be estimated as a deviation, the knee point, from a linear rise, independent of wavefront direction. **(B)** Heatmap of CV values at the ablated area center of rat atria as a function of RFA duration and CM radius. The heatmap shows the expansion of the ablated area with each subsequent ablation step characterized by distinctive CV slowing. (Bottom) CV curves are shown for different RFA steps, with a knee point for the approximate radius of 25 pixels, or 2 mm. **(C)** Whole isolated guinea pig heart. The CV curve at the center of ablation (RFA of 3 s) for different CM radii flattens for an approximate radius of 2.5 mm, indicating an RFA-created lesion of 5 mm diameter. **(D)** Human heart epicardium of LV. From the figure, the CV curve, obtained in the same fashion, flattens for an approximate radius of 1.5 mm. **(E)** Human heart endocardium of LV. Upper, for deep-red light excitation, the CV curve flattens for an approximate CM radius of 2.5 mm, and CM radius of 4 mm for green light excitation (lower figure), indicating narrowing of RFA-created lesion across the transmural wall.

Although the ablated tissue is not excitable, a signal resembling AP can be measured within the ablated area due to electronic coupling between the excitable cardiac cells and a lesion consisting of unexcitable cells (Quinn et al., 2016). APs inside the lesion show characteristic distorted morphology (Figure 3D) and decreased amplitude (Figure 5E), characteristic of electrotonic coupling. As electrotonic coupling slows wavefront propagation, the ablated region size can be characterized as a CV decrease. The numerical simulations show CV linearly decreases inside the ablation zone toward the ablation center. This allows easy estimation of the lesion size as a knee point deviation from linear CV rise from the ablation center (Figure 7A). The same pattern was observed in experiments. The CV increases linearly with the CM radius increase, centered inside the lesion, as long as the radius is smaller than the ablated area (Figure 7). The knee points of curves shown in Figure 7 estimate ablated areas size very well. For example, the CV vs. radius curve for guinea pig ventricular ablation estimates diameter of the ablated area of 5 mm, which is in agreement with the ΔCV map shown in Figure 4C for RFA of 3 s. The limitation of this approach is circular-shaped approximation of the ablated area; as for elliptically shaped lesions, the knee point corresponds to the minor axis.

A distinct feature of the proposed CM is its inherent adaptation to the local changes in a propagating wavefront direction, making the method independent of the propagating wavefront direction. CV maps are also obtained through automatized data processing, identifying the wavefront propagation direction upon which the CV’s magnitude and angle are obtained. The CM accuracy depends on the chosen circle radius approximating planar wavefront propagation inside the circle. With increased radius, the accuracy decreases to estimate a true local CV. However, the precision increases as LAT differences are calculated along chords spanning larger spatial differences. In experimental setups, the minimum radius of 1 mm was chosen, to balance the accuracy and precision, resulting in CV maps resolving small conduction heterogeneities with acceptable SNR.

### 4.1 Comparison With Other CV Methods

In general, optical mapping-based methods estimating local CV differ due to particular study goals. The gold standard for measuring conduction properties (Bayly et al., 1998) is based on a polynomial surface fit over a LAT map to estimate local gradients, whose implementation is available as the Rhythm MATLAB toolkit (Gloschat et al., 2018). However, this method is not ideally suited for heterogeneously conducting tissue, or for those whose activation does not have a continuous pattern, as with calcium imaging in cell cultures. The Ccoffinn method (Tomek et al., 2016) has been developed as an alternative to the standard polynomial surface fit method. The method considers wavefronts from sequential frames, using a graph-based algorithm to find a set of vectors that best describe the direction and velocity of wave propagation. Tissue anisotropy is commonly studied following an activation wavefront spread starting from the central pacing site. Single vector and average vector method (Linnenbank et al., 2014) approaches are helpful to estimate tissue anisotropy by quantifying longitudinal and transversal CV, assuming a homogeneously anisotropic myocardium, and the methods are implemented in ElectroMap open source for analysis of cardiac electrophysiology (O’Shea et al., 2019). The assumption that transverse propagation lies perpendicular to the longitudinal does not consider the influence of irregularity of tissue geometry or heterogeneous discontinuities. The semi-automated ORCA method has been developed to study tissue anisotropy addressing the issue of non-orthogonality (Doshi et al., 2015) to obtain longitudinal and transversal CV and is implemented in the Rhythm toolkit. ORCA employs the single vector method assuming that the CV is constant in an anisotropic 2D sheet at a distance from the pacing site. Longitudinal and transversal CVs were estimated using the linear fits on segments of activation time curves along different directions spanning a complete circle to detect the maximal and minimal slopes of the linear fits corresponding to longitudinal and transverse CVs.

In clinical practice, triangulation is commonly used among many different CV methods, as the method is not constrained by electrode configurations and is suitable for mapping catheters of different electrode configurations (Cantwell et al., 2014). The accuracy of the triangulation method depends on the size of a selected triangle, as the CV values are interpolated inside the triangle assuming locally planar wavefront propagation (Cantwell et al., 2015). FiD method is based on a 2D grid layout of measurement points, determining the difference in activation times with known spatial distance between neighboring points and two orthogonal directions, and is suitable for optical mapping measurement. As the CV is calculated using a global coordinate system, the accuracy of the method depends on the wavefront propagating direction. For example, the same activation time of neighboring points results in large CV uncertainties or unphysiological high CV.

The cosine fit is another CV method explicitly developed for atrial mapping. As the arrangement of measurement electrodes in the clinical environment depends on the choice of a catheter, the cosine fit approach (Weber et al., 2010) allows estimation of local CV and the angle of incidence from measured electrograms using electrodes positioned on a circular catheter under sinus or pace mapping. This single-shot analysis is suitable for determining the pacing source, assuming a sufficiently large distance from the wavefront source and assuming planar wavefront propagation inside the circular catheter. A limitation of this analysis is that the locally determined angle of incidence does not necessarily point toward the stimulation origin. As wavefronts may exhibit curvature, particularly if originating from a nearby focal source, the improved cosine-fit method (Roney et al., 2014) allows CV determination and the direction toward the location of a focal source for either circular or planar wavefronts, recorded from the arbitrary arrangements of electrodes. As both methods assume isotropic conductivity to estimate a focal source to address tissue conduction anisotropy and heterogeneities, another cosine-fit-type method (Roney et al., 2018) combines multiple activation maps from different pacing. This technique is suitable for estimating conduction anisotropy and fiber direction from clinically available atrial electrical recordings.

We did not quantitatively compare cosine-based methods with the CM to test for accuracy, as both are developed for different purposes. Cosine-based methods are developed for clinical atrial mapping applications to efficiently extract patient-specific substrate parameters for patients undergoing ablation. CM is easy to implement in optical mapping-based ventricular and atrial data experiments to characterize ablated lesions. CM applicability can be extended to determine focal sources, as propagating wavefront direction changes can be tracked. However, optical mapping is currently limited to basic science research.

**FIGURE 8:**
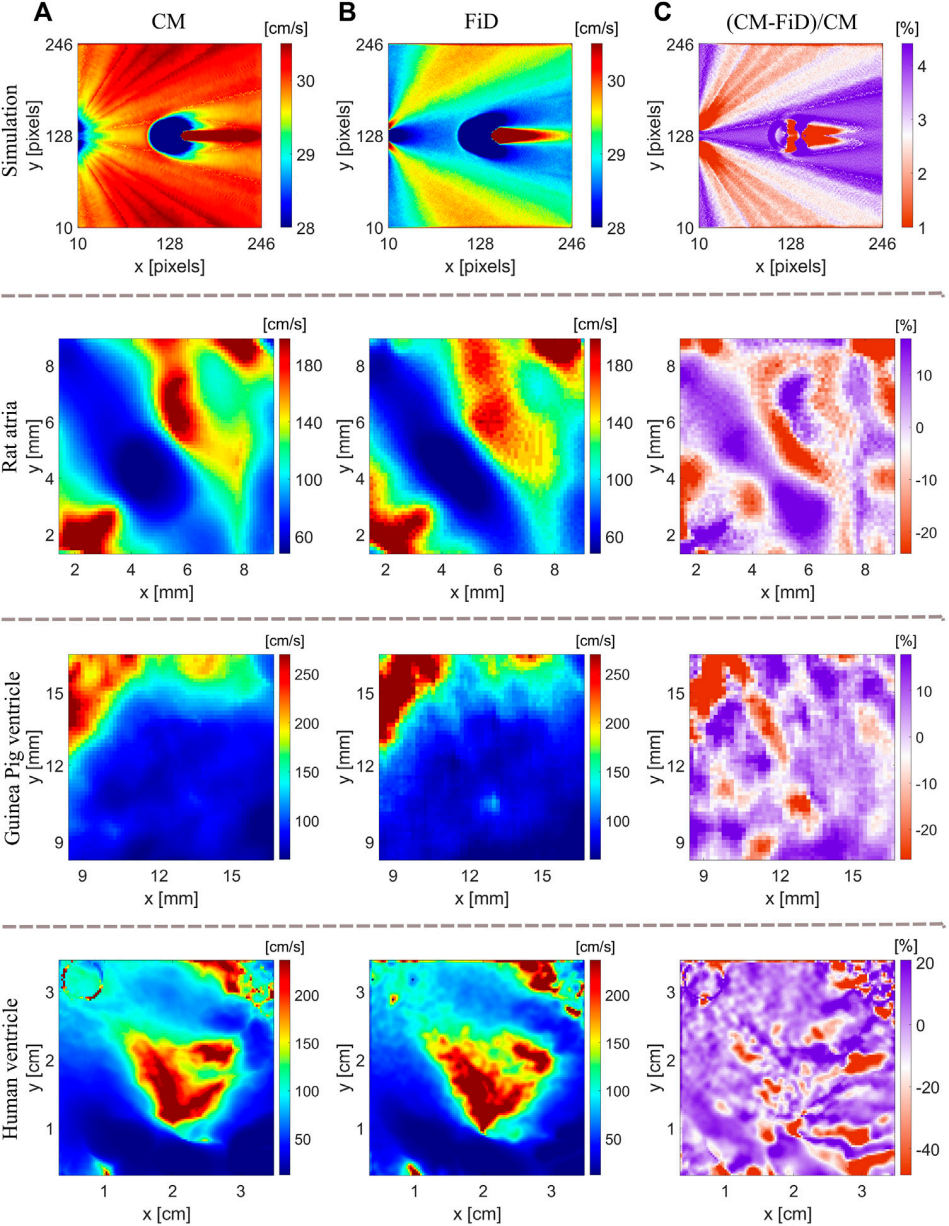
Comparison of CV maps using the CM and FiD method. **(A)** CV maps generated with the CM method for the simulation, rat’s atria, guinea pig’s ventricle, and human ventricle (epicardium). **(B)** Respective CV maps obtained with the FiD method. **(C)** Relative difference of CV maps obtained with the two methods.

The CM method can be viewed as an extension of the FiD method as both methods are based on LAT differences over a 2D grid, and the two methods were compared using both numerical and experimental data (Figures 8A,B). The relative CV difference between the two methods, (CM-FiD)/CM, is shown in Figure 8C. As expected with numerical data, both methods result in similar CV maps with small relative differences. The disparity between the two methods increases significantly for experimental data, ranging from −20% to 10% for rat atria and isolated guinea pig hearts and −40% and 20% for highly trabeculated human ventricular endocardium. We also investigated whether the choice of radius plays a role in the observed differences. We compared the CV maps obtained with the two methods for radius ranging from 5 to 10 pixels. However, the differences between the two methods were consistent. With CM, the local CV is calculated using a locally defined coordinate system with one of the axes coaligning with the automatically identified wavefront direction. In contrast, FiD is based on a globally defined coordinate system, obtaining x and y components separately. If a propagating wavefront line aligns with one of the axes, LAT differences can be zero, resulting in large CV uncertainties. In Figure 8B, the FiD overestimates CV values in rat atria for the red-colored region in the middle of the CV map. Since the wavefront is changing its direction and moving down vertically in some parts of the atria (Figure 3A), large uncertainties of CV *x*-axis component result in a rapid rise of local CV.

One of the challenges in ventricular tissue ablation is depth estimation of the created lesions. In this study, we used two different *V*_*m*_ dye excitation bands, a green band centered at 525 nm and a deep red band centered around 660 nm, for optical mapping of the human endocardium. While no measurement of penetration depth for different wavelengths is performed, penetration depth in the skin is generally under 1 mm for the green light and over 3 mm for deep red light (Avci et al., 2013). Using two different light bands allowed us to study the differences in CV maps obtained from AP signals emanating from the surface layer on the epicardial side and AP signals from the deeper layers (Figure 6D). A highly trabeculated endocardium of the human left ventricle and the presence of papillary muscle result in a highly heterogeneous CV map which would hinder the ablated area as the zone of decreased CV (Figures 6B,C). Amplitude alternans, the beat-to-beat variation in AP amplitudes, occur when the dispersion of AP refractoriness results in decreased excitability and can be quantified as a decrease in CV. As shown in Supplemental Figure S2, the depth of the created lesion is 3–4 mm, measured optical AP traces from the surface layer with green light excitation are solely due to electrotonic coupling between excitable cells and a lesion, resulting in no alternans at PCL of 800 ms. However, deep-red-light excitation shows the presence of CV alternans due to incomplete transmural ablation. One mechanism leading to CV alternans could be due to the presence of unexcitable cells coupled with excitable cells resulting in reduced cell-to-cell coupling, leading to decreased excitability.

## 5 Conclusion

In this study, RFA-created lesions were quantified as local CV decrease using optical mapping measurements with submillimeter spatial resolution. For the application of the CM method, no prior information about the propagating wavefront direction is required, and CM robustness was tested and validated in numerical simulations, and experimentally on isolated rat’s atria, whole guinea pig’s heart, and human hearts. RFA-created lesion profiles were quantified as the relative change of CV before and after ablation or, as in the case of a human heart with highly trabeculated endocardium, by analyzing CV alternans using excitation light bands of different penetration depths to estimate lesion depth across the thick transmural wall. As the CV is one of the important parameters for studying heart electrophysiology, CM can be applied to other studies, such as identifying slow zones for mapping VT substrates.

The histopathological features of RFA have been mainly studied in normal myocardia, and its effect on clinically relevant heterogeneous scars is not well understood. For treatment of reentrant VT, reentry typically occurs within the scarred region due to tissue heterogeneities, and the RFA prevents reentry by homogenizing the scared areas. However, studies show that scar tissue is more resistant to thermal injury compared to healthy cardiomyocytes (Barkagan et al., 2019), which may impair the effectiveness of the RFA procedure. Optical mapping with near-infrared *V*_*m*_ dyes enables measurements of transmural wavefront propagation (Herndon et al., 2016; Uzelac et al., 2016) for better depth characterization of the scar area. Although limited to lab research, the high spatial resolution of optical mapping enables quantitatively predictive studies of how local CV changes affect heart electrophysiology. New findings may add toward a better understanding of arrhythmia mechanisms and ablation effects on tissue electrophysiology to develop improved ablation strategies (Jalife et al., 2002; Sanders et al., 2005; Narayan et al., 2012; Clayton and Nash, 2015; Hansen et al., 2015).

## 6 Limitations

In this study, experiments were performed at different institutions. Histological evaluations were not performed. Statistical analysis was not possible due to the limited number of experiments. Nevertheless, one of the main objectives of this study is to present the method and results suitable for further investigation of RFA outcome assessment.

## Data Availability

The raw data supporting the conclusions of this article will be made available by the authors upon reasonable request.

## References

[B1] Allessie M. A. Bonke F. I. Schopman F. J. (1977). Circus movement in rabbit atrial muscle as a mechanism of tachycardia. III. The "leading circle" concept: a new model of circus movement in cardiac tissue without the involvement of an anatomical obstacle. Circ. Res. 41, 9–18. 10.1161/01.res.41.1.9 862147

[B2] Allessie M. de Groot N. (2014). CrossTalk opposing view: Rotors have Not been demonstrated to be the drivers of atrial fibrillation. J. Physiol. 592, 3167–3170. 10.1113/jphysiol.2014.271809 25085969 PMC4146363

[B3] Avci P. Gupta A. Sadasivam M. Vecchio D. Pam Z. Pam N. (2013). Low-level laser (light) therapy (lllt) in skin: stimulating, healing, restoring. Semin. Cutan. Med. Surg. 32, 41–52. 24049929 PMC4126803

[B4] Aziz Z. Shatz D. Raiman M. Upadhyay G. A. Beaser A. D. Besser S. A. (2019). Targeted Ablation of Ventricular Tachycardia Guided by Wavefront Discontinuities During Sinus Rhythm. Circulation 140, 1383–1397. 10.1161/CIRCULATIONAHA.119.042423 31533463

[B5] Barkagan M. Leshem E. Shapira-Daniels A. Sroubek J. Buxton A. E. Saffitz J. E. (2019). Histopathological characterization of radiofrequency ablation in ventricular scar tissue. JACC Clin. Electrophysiol. 5, 920–931. 10.1016/j.jacep.2019.05.011 31439293

[B6] Bastos-Filho T. F. (2021). XXVII Brazilian Congress on Biomedical Engineering, Vol. 83. Berlin, Germany: Springer Nature.

[B7] Bayly P. V. KenKnight B. H. Rogers J. M. Hillsley R. E. Ideker R. E. Smith W. M. (1998). Estimation of conduction velocity vector fields from epicardial mapping data. IEEE Trans. Biomed. Eng. 45, 563–571. 10.1109/10.668746 9581054

[B8] Benjamin E. J. Muntner P. Alonso A. Bittencourt M. S. Callaway C. W. Carson A. P. (2019). Heart Disease and Stroke Statistics-2019 Update: A Report From the American Heart Association. Circulation 139, e56–e528. 10.1161/CIR.0000000000000659 30700139

[B9] Berenfeld O. Ennis S. Hwang E. Hooven B. Grzeda K. Mironov S. (2011). Time- and frequency-domain analyses of atrial fibrillation activation rate: The optical mapping reference. Heart rhythm. 8, 1758–1765. 10.1016/j.hrthm.2011.05.007 21699849 PMC3202688

[B10] Cantwell C. D. Roney C. H. Ali R. L. Qureshi N. A. Lim P. B. Peters N. S. (2014). A software platform for the comparative analysis of electroanatomic and imaging data including conduction velocity mapping. Annu. Int. Conf. IEEE Eng. Med. Biol. Soc. 2014, 1591–1594. 10.1109/EMBC.2014.6943908 25570276

[B11] Cantwell C. D. Roney C. H. Ng F. S. Siggers J. H. Sherwin S. J. Peters N. S. (2015). Techniques for automated local activation time annotation and conduction velocity estimation in cardiac mapping. Comput. Biol. Med. 65, 229–242. 10.1016/j.compbiomed.2015.04.027 25978869 PMC4593301

[B12] Chen D. D. Gray R. A. Uzelac I. Herndon C. Fenton F. H. (2017). Mechanism for amplitude alternans in electrocardiograms and the initiation of spatiotemporal chaos. Phys. Rev. Lett. 118, 168101. 10.1103/physrevlett.118.168101 28474934

[B13] Clayton R. H. Nash M. P. (2015). Analysis of cardiac fibrillation using phase mapping. Card. Electrophysiol. Clin. 7, 49–58. 10.1016/j.ccep.2014.11.011 25784022

[B14] Dice L. R. (1945). Measures of the amount of ecologic association between species. Ecology 26, 297–302. 10.2307/1932409

[B15] Doshi A. N. Walton R. D. Krul S. P. de Groot J. R. Bernus O. Efimov I. R. (2015). Feasibility of a semi-automated method for cardiac conduction velocity analysis of high-resolution activation maps. Comput. Biol. Med. 65, 177–183. 10.1016/j.compbiomed.2015.05.008 26045101

[B16] Efimov I. R. Nikolski V. P. Salama G. (2004). Optical imaging of the heart. Circulation Res. 95, 21–33. 10.1161/01.res.0000130529.18016.35 15242982

[B17] El-Sherif N. Gough W. B. Restivo M. (1987). Reentrant ventricular arrhythmias in the late myocardial infarction period: 14. mechanisms of resetting, entrainment, acceleration, or termination of reentrant tachycardia by programmed electrical stimulation. Pacing Clin. Electrophysiol. 10, 341–371. 10.1111/j.1540-8159.1987.tb05974.x 2437540

[B18] Fedorov V. V. Schuessler R. B. Hemphill M. Ambrosi C. M. Chang R. Voloshina A. S. (2009). Structural and functional evidence for discrete exit pathways that connect the canine sinoatrial node and atria. Circulation Res. 104, 915–923. 10.1161/circresaha.108.193193 19246679 PMC2740656

[B19] Fenton F. Karma A. (1998). Vortex dynamics in three-dimensional continuous myocardium with fiber rotation: Filament instability and fibrillation. Chaos 8, 20–47. 10.1063/1.166311 12779708

[B20] Foppen S. (2009). Experimental and Numerical Analysis of Lesion Growth during Cardiac Radiofrequency Ablation

[B21] Fukumoto K. Habibi M. Ipek E. G. Zahid S. Khurram I. M. Zimmerman S. L. (2016). Association of left atrial local conduction velocity with late gadolinium enhancement on cardiac magnetic resonance in patients with atrial fibrillation. Circulation Arrhythmia Electrophysiol. 9, e002897. 10.1161/circep.115.002897 PMC4772170 26917814

[B22] Gerig G. Kubler O. Kikinis R. Jolesz F. A. (1992). Nonlinear anisotropic filtering of mri data. IEEE Trans. Med. Imaging 11, 221–232. 10.1109/42.141646 18218376

[B23] Gloschat C. Aras K. Gupta S. Faye N. R. Zhang H. Syunyaev R. A. (2018). Rhythm: an open source imaging toolkit for cardiac panoramic optical mapping. Sci. Rep. 8, 2921. 10.1038/s41598-018-21333-w 29440763 PMC5811559

[B24] Haines D. E. (1993). The biophysics of radiofrequency catheter ablation in the heart: The importance of temperature monitoring. Pacing Clin. Electro 16, 586–591. 10.1111/j.1540-8159.1993.tb01630.x 7681962

[B25] Hansen B. J. Zhao J. Csepe T. A. Moore B. T. Li N. Jayne L. A. (2015). Atrial fibrillation driven by micro-anatomic intramural re-entry revealed by simultaneous sub-epicardial and sub-endocardial optical mapping in explanted human hearts. Eur. Heart J. 36, 2390–2401. 10.1093/eurheartj/ehv233 26059724 PMC4568403

[B26] Herndon C. Uzelac I. Farmer J. T. Fenton F. (2016). “Computational ecg reconstruction and validation from high-resolution optical mapping,” in 2016 Computing in Cardiology Conference (CinC) (Vancouver, BC, Canada: IEEE), 713–716. 10.22489/cinc.2016.208-518

[B27] Hong K. L. Redfearn D. Chacko S. Baley J. Baranchuk A. Glover B. M. (2019). High-resolution mapping of the atria using the hd grid catheter. Hear. Case Rep. 5, 351–353. 10.1016/J.HRCR.2018.12.012 PMC6630112 31341774

[B28] Irie T. Yu R. Bradfield J. S. Vaseghi M. Buch E. F. Ajijola O. (2015a). Relationship between sinus rhythm late activation zones and critical sites for scar-related ventricular tachycardia. Circ Arrhythmia Electrophysiol. 8, 390–399. 10.1161/CIRCEP.114.002637 PMC4695215 25740836

[B29] Irie T. Yu R. Bradfield J. S. Vaseghi M. Buch E. F. Ajijola O. (2015b). Relationship Between Sinus Rhythm Late Activation Zones and Critical Sites for Scar-Related Ventricular Tachycardia. Circ Arrhythmia Electrophysiol. 8, 390–399. 10.1161/circep.114.002637 PMC4695215 25740836

[B30] Jalife J. Berenfeld O. Mansour M. (2002). Mother rotors and fibrillatory conduction: a mechanism of atrial fibrillation. Cardiovasc. Res. 54, 204–216. 10.1016/s0008-6363(02)00223-7 12062327

[B31] Kim Y. H. Chen S. A. Ernst S. Guzman C. E. Han S. Kalarus Z. (2020). 2019 APHRS expert consensus statement on three‐dimensional mapping systems for tachycardia developed in collaboration with HRS, EHRA, and LAHRS. J. Arrhythmia 36, 215–270. 10.1002/joa3.12308 PMC7132207 32256872

[B32] Kuo C. S. Munakata K. Reddy C. P. Surawicz B. (1983). Characteristics and possible mechanism of ventricular arrhythmia dependent on the dispersion of action potential durations. Circulation 67, 1356–1367. 10.1161/01.cir.67.6.1356 6851031

[B33] Lalani G. G. Schricker A. Gibson M. Rostamian A. Krummen D. E. Narayan S. M. (2012). Atrial Conduction Slows Immediately Before the Onset of Human Atrial Fibrillation. J. Am. Coll. Cardiol. 59, 595–606. 10.1016/j.jacc.2011.10.879 22300695 PMC3390156

[B34] Linnenbank A. C. de Bakker J. M. T. Coronel R. (2014). How to measure propagation velocity in cardiac tissue: a simulation study. Front. Physiol. 5. 10.3389/FPHYS.2014.00267 PMC4106028 25101004

[B35] Malaczynska-Rajpold K. Blaszyk K. Kociemba A. Pyda M. Posadzy-Malaczynska A. Grajek S. (2020). Islets of heterogeneous myocardium within the scar in cardiac magnetic resonance predict ventricular tachycardia after myocardial infarction. J. Cardiovasc Electrophysiol. 31, 1452–1461. 10.1111/jce.14461 32227520

[B36] Mironov S. Jalife J. Tolkacheva E. G. (2008). Role of conduction velocity restitution and short-term memory in the development of action potential duration alternans in isolated rabbit hearts. Circulation 118, 17–25. 10.1161/CIRCULATIONAHA.107.737254 18559701 PMC2574713

[B37] Moe G. K. Rheinboldt W. C. Abildskov J. A. (1964). A computer model of atrial fibrillation. Am. Heart J. 67, 200–220. 10.1016/0002-8703(64)90371-0 14118488

[B38] Narayan S. M. Franz M. R. Clopton P. Pruvot E. J. Krummen D. E. (2011). Repolarization alternans reveals vulnerability to human atrial fibrillation. Circulation 123, 2922–2930. 10.1161/circulationaha.110.977827 21646498 PMC3135656

[B39] Narayan S. M. Krummen D. E. Shivkumar K. Clopton P. Rappel W.-J. Miller J. M. (2012). Treatment of Atrial Fibrillation by the Ablation of Localized Sources. J. Am. Coll. Cardiol. 60, 628–636. 10.1016/j.jacc.2012.05.022 22818076 PMC3416917

[B40] Nattel S. Shiroshita-Takeshita A. Brundel B. J. J. M. Rivard L. (2005). Mechanisms of atrial fibrillation: lessons from animal models. Prog. Cardiovasc. Dis. 48, 9–28. 10.1016/j.pcad.2005.06.002 16194689

[B41] Ng F. S. Holzem K. M. Koppel A. C. Janks D. Gordon F. Wit A. L. (2014). Adverse Remodeling of the Electrophysiological Response to Ischemia-Reperfusion in Human Heart Failure Is Associated With Remodeling of Metabolic Gene Expression. Circ Arrhythmia Electrophysiol. 7, 875–882. 10.1161/circep.113.001477 PMC4206603 25114062

[B42] O'Shea C. Holmes A. P. Yu T. Y. Winter J. Wells S. P. Correia J. (2019). Electromap: high-throughput open-source software for analysis and mapping of cardiac electrophysiology. Sci. Rep. 9, 1389. 10.1038/s41598-018-38263-2 30718782 PMC6362081

[B43] Perona P. Malik J. (1990). Scale-space and edge detection using anisotropic diffusion. IEEE Trans. Pattern Anal. Mach. Intell. 12, 629–639. 10.1109/34.56205

[B44] Pollnow S. (2018). Characterizing Cardiac Electrophysiology during Radiofrequency Ablation, Vol. 24. Karlsruhe, Germany: Karlshure Transactions on Biomedical Engineering.

[B45] Prystowsky E. N. (2008). The history of atrial fibrillation: The last 100 years. J. Cardiovasc. Electrophysiol. 19, 575–582. 10.1111/j.1540-8167.2008.01184.x 18462324

[B46] Quinn T. A. Camelliti P. Rog-Zielinska E. A. Siedlecka U. Poggioli T. O'Toole E. T. (2016). Electrotonic coupling of excitable and nonexcitable cells in the heart revealed by optogenetics. Proc. Natl. Acad. Sci. U.S.A. 113, 14852–14857. 10.1073/pnas.1611184114 27930302 PMC5187735

[B47] Rensma P. L. Allessie M. A. Lammers W. J. Bonke F. I. Schalij M. J. (1988). Length of excitation wave and susceptibility to reentrant atrial arrhythmias in normal conscious dogs. Circ. Res. 62 (2), 395–410. 10.1161/01.res.62.2.395 3338122

[B48] Roney C. H. Cantwell C. D. Qureshi N. A. Ali R. L. Chang E. T. Lim P. B. (2014). An automated algorithm for determining conduction velocity, wavefront direction and origin of focal cardiac arrhythmias using a multipolar catheter. Annu. Int. Conf. IEEE Eng. Med. Biol. Soc. 2014, 1583–1586. 10.1109/EMBC.2014.6943906 25570274

[B49] Roney C. Whitaker J. Sim I. O’Neill L. Mukherjee R. Razeghi O. (2018). A technique for measuring anisotropy in atrial conduction to estimate conduction velocity and atrial fibre direction. Comput. Biol. Med. 104, 278–290. 10.1016/j.compbiomed.2018.10.019 30415767 PMC6506689

[B50] Rudy Y. (2012). Comprehensive Biophysics.

[B51] Sanders P. Berenfeld O. Hocini M. Jaïs P. Vaidyanathan R. Hsu L.-F. (2005). Spectral analysis identifies sites of high-frequency activity maintaining atrial fibrillation in humans. Circulation 112, 789–797. 10.1161/circulationaha.104.517011 16061740

[B52] Sasaki T. Calkins H. Miller C. F. Zviman M. M. Zipunnikov V. Arai T. (2015). New insight into scar-related ventricular tachycardia circuits in ischemic cardiomyopathy: Fat deposition after myocardial infarction on computed tomography-A pilot study. Heart rhythm. 12, 1508–1518. 10.1016/j.hrthm.2015.03.041 25814415 PMC4485604

[B53] Shinagawa K. Mitamura H. Takeshita A. Sato T. Kanki H. Takatsuki S. (2000). Determination of refractory periods and conduction velocity during atrial fibrillation using atrial capture in dogs. J. Am. Coll. Cardiol. 35, 246–253. 10.1016/S0735-1097(99)00488-X 10636287

[B54] Spector P. (2013). Principles of cardiac electric propagation and their implications for re-entrant arrhythmias. Circ Arrhythmia Electrophysiol. 6, 655–661. 10.1161/circep.113.000311 23778249

[B55] Takeuchi S. Akita T. Takagishi Y. Watanabe E. Sasano C. Honjo H. (2006). Disorganization of gap junction distribution in dilated atria of patients with chronic atrial fibrillation. Circ. J. 70, 575–582. 10.1253/circj.70.575 16636493

[B56] Tan V. H. Lyu M. Z. Tan P. C. Wong L. C. Yeo C. Wong K. C. K. (2020). Utility of directional high‐density mapping catheter (Advisor TM HD Grid) in complex scar‐related atrial tachycardia. J. Arrhythmia 36, 180–183. 10.1002/JOA3.12256 PMC7011828 32071639

[B57] Thompson S. A. Copeland C. R. Reich D. H. Tung L. (2011). Mechanical coupling between myofibroblasts and cardiomyocytes slows electric conduction in fibrotic cell monolayers. Circulation 123, 2083–2093. 10.1161/circulationaha.110.015057 21537003 PMC3176459

[B58] Tomek J. Burton R. A. B. Bub G. (2016). Ccoffinn: Automated wave tracking in cultured cardiac monolayers. Biophysical J. 111, 1595–1599. 10.1016/j.bpj.2016.08.049 PMC5072981 27760347

[B59] Tu Y. X. Wernsdörfer A. Honda S. Tomita Y. (1997). Estimation of conduction velocity distribution by regularized-least-squares method. IEEE Trans. Biomed. Eng. 44, 1102–1106. 10.1109/10.641337 9353989

[B60] Uzelac I. Herndon C. Farmer J. Fenton F. (2016). Electrocardiogram reconstruction from high resolution voltage optical mapping. Annu. Int. Conf. IEEE Eng. Med. Biol. Soc. 2016, 3941–3944. 10.1109/EMBC.2016.7591589 28269147

[B61] Uzelac I. Fenton F. (2015). “Robust framework for quantitative analysis of optical mapping signals without filtering,” in 2015 Computing in Cardiology Conference (CinC) (Nice, France: IEEE), 461–464. 10.1109/cic.2015.7408686

[B62] Uzelac I. Iravanian S. Fenton G. (2019). “Parallel acceleration on removal of optical mapping baseline wandering,” in 2017 Computing in Cardiology (CinC) (Singapore: IEEE), 1–4. 10.22489/cinc.2019.433 PMC9202644 35719209

[B63] Uzelac I. Ji Y. C. Hornung D. Schröder-Scheteling J. Luther S. Gray R. A. (2017). Simultaneous quantification of spatially discordant alternans in voltage and intracellular calcium in langendorff-perfused rabbit hearts and inconsistencies with models of cardiac action potentials and ca transients. Front. Physiol. 8, 819. 10.3389/fphys.2017.00819 29104543 PMC5655020

[B64] Uzelac I. Kaboudian A. Iravanian S. Siles-Paredes J. G. Gumbart J. C. Ashikaga H. (2021). Quantifying arrhythmic long qt effects of hydroxychloroquine and azithromycin with whole-heart optical mapping and simulations. Heart Rhythm O2 2, 394–404. 10.1016/j.hroo.2021.06.008 34430945 PMC8369304

[B65] van Schie M. S. Heida A. Taverne Y. J. H. J. Bogers A. J. J. C. de Groot N. M. S. (2021). Identification of local atrial conduction heterogeneities using high-density conduction velocity estimation. EP Eur. 23, 1815–1825. 10.1093/europace/euab088 PMC8576284 33970234

[B66] Vasquez C. Mohandas P. Louie K. L. Benamer N. Bapat A. C. Morley G. E. (2010). Enhanced Fibroblast-Myocyte Interactions in Response to Cardiac Injury. Circ. Res. 107, 1011–1020. 10.1161/circresaha.110.227421 20705922 PMC2993566

[B67] Walton R. D. Smith R. M. White E. Bernus O. Pertsov A. M. Pertsov A. M. (2012). Extracting surface activation time from the optically recorded action potential in three-dimensional myocardium. Biophysical J. 102(1), 30–38. 10.1016/j.bpj.2011.10.036 PMC3250680 22225795

[B68] Weber F. M. Schilling C. Seemann G. Luik A. Schmitt C. Lorenz C. (2010). Wave-Direction and Conduction-Velocity Analysis From Intracardiac Electrograms-A Single-Shot Technique. IEEE Trans. Biomed. Eng. 57, 2394–2401. 10.1109/tbme.2010.2055056 20595079

[B69] Wittkampf F. H. M. Nakagawa H. (2006). Rf catheter ablation: lessons on lesions. Pacing Clin. Electro 29, 1285–1297. 10.1111/j.1540-8159.2006.00533.x 17100685

[B70] Zaman J. A. B. Baykaner T. Schricker A. A. Krummen D. E. Narayan S. M. (2017). Mechanistic targets for the ablation of atrial fibrillation. Glob. Cardiol. Sci. Pract. 2017, e201707. 10.21542/gcsp.2017.7 28971106 PMC5621726

[B71] Zimerman L. Fenelon G. Martinelli M. (2009). Diretrizes Brasileiras de Fibrilação Atrial, vol. 92(6 supl. 1) (Arq Bras Cardiol: Sociedade Brasileira de Cardiologia).

