# Supplementary figures and images for: Circle Method for Robust Estimation of Local Conduction Velocity High-Density Maps From Optical Mapping Data: Characterization of Radiofrequency Ablation Sites
Source: Front Physiol. 2022 Aug 12;13:794761. doi: 10.3389/fphys.2022.794761 (PMC9417315; doi:10.3389/fphys.2022.794761)

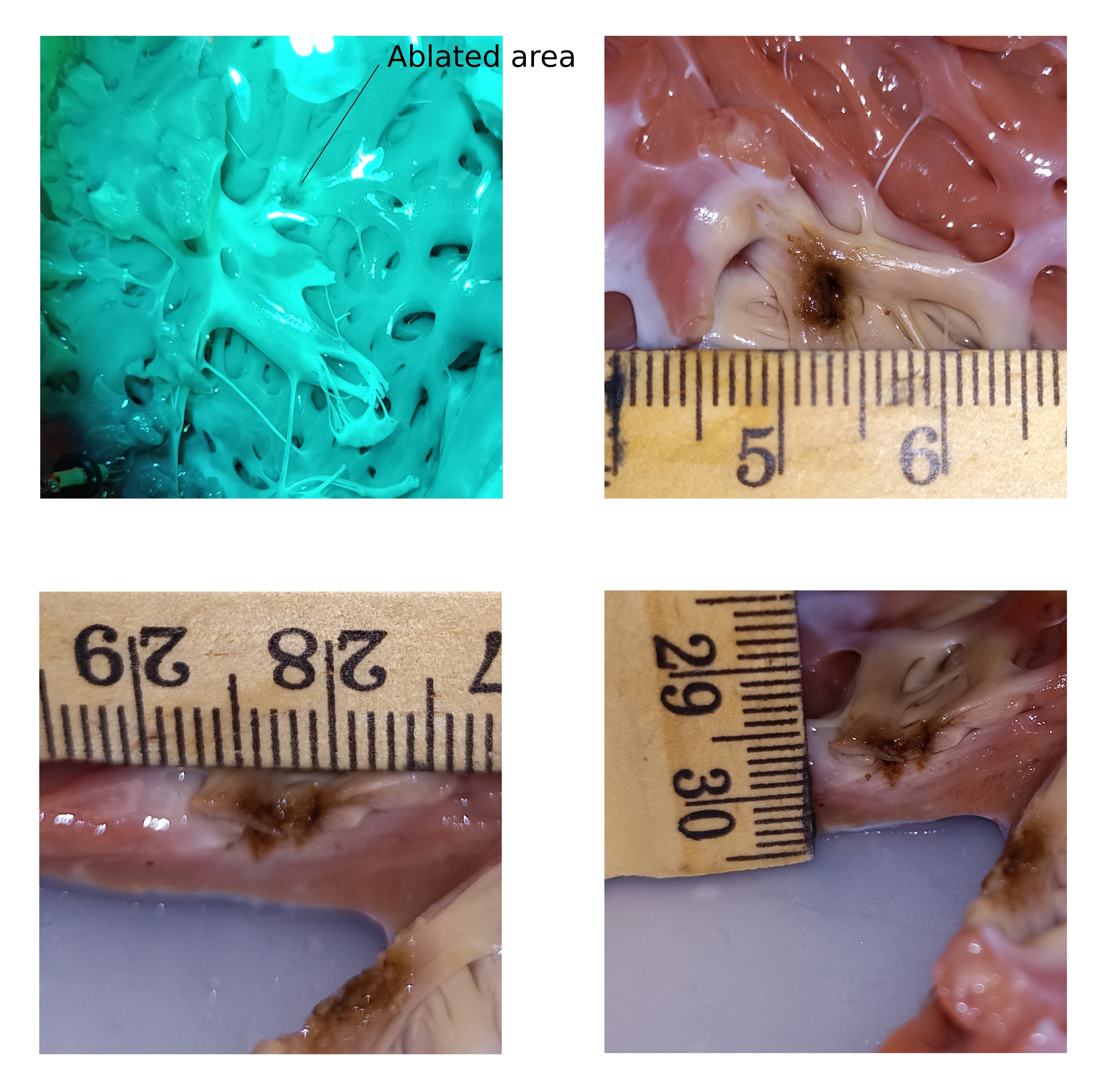

Supplement: Supplementary file 1 [file Image2.JPEG]

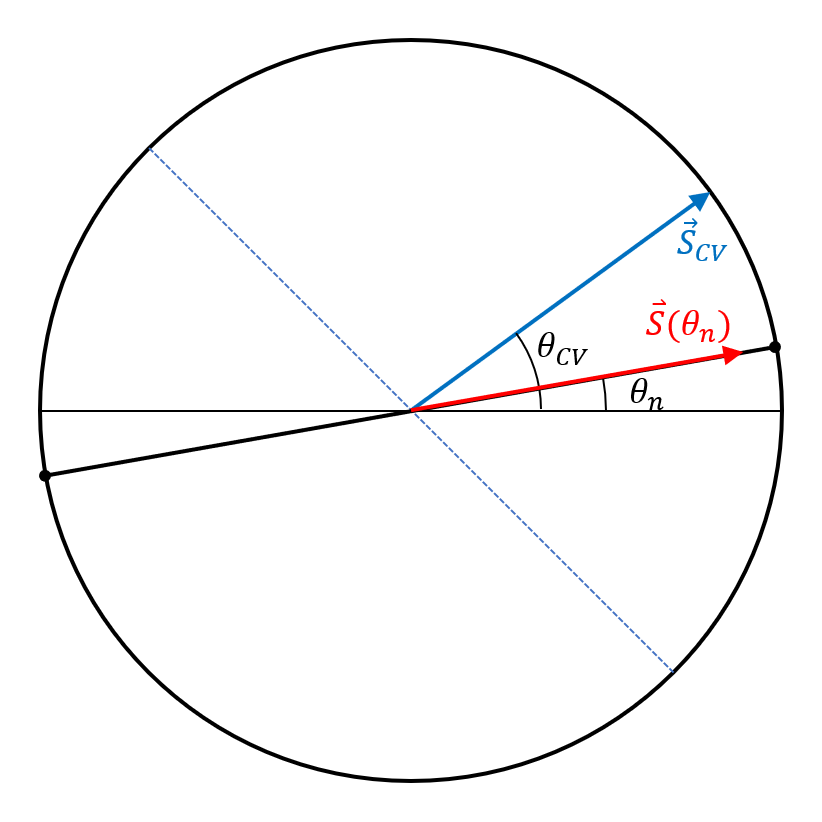

Supplement: Supplementary file 3 [file Image1.PNG]
