# Supplementary material for: Circle Method for Robust Estimation of Local Conduction Velocity High-Density Maps From Optical Mapping Data: Characterization of Radiofrequency Ablation Sites
Source: Front Physiol. 2022 Aug 12;13:794761. doi: 10.3389/fphys.2022.794761 (PMC9417315; doi:10.3389/fphys.2022.794761)
Supplement: Supplementary file 2 [file DataSheet1.PDF]

Supplemental Materials

Correspondence*:
jimena.gabriela@ufabc.edu.br

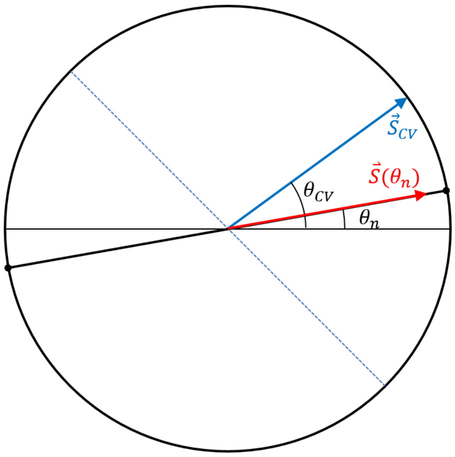

Supplemental Figure 1. Illustration of a plane wave's conduction speed being calculated from the circle method. A plane wave (blue dotted line) is propagating along the $\vec{S}_{CV}$ direction. A circle is defined around a point on the wavefront, and time differences across a chord oriented along θ_n is used to calculate conduction speed along this test chord, $\vec{S}(\theta_n)$.

If a plane wave is propagating along the $\vec{S}_{CV}$ direction (illustrated in Figure 1), and the speed is calculated from a time difference, Δt, along the chord at θ_n (i.e. $S(\theta_n) = 2R/\Delta t$), the speed will be reported as slower than the true speed. Specifically, the speed measured along this chord would be

$$\begin{aligned} |\vec{S}(\theta_n)| &= \vec{S}_{CV} \cdot \hat{r}(\theta_n) \\ &= |\vec{S}_{CV}| \cos{(\theta_{CV} - \theta_n)}. \end{aligned}$$

Therefore, if the speed measurement was across a chord oriented along θ_n, than $|\vec{S}_{CV}|$ could be calculated as

$$\begin{aligned} |\vec{S}_{CV}| &= \frac{S(\theta_n)}{\cos{(\theta_{CV} - \theta_n)}} \\ &\approx \frac{S(\theta_n)}{\cos{(\theta_{PD} - \theta_n)}}. \end{aligned} \quad (1)$$

Since the circle is discretized, the true propagation direction is approximated with the closest chord along θ_{CV}, which is denoted θ_{PD}. As the number of chords increases, this approximation becomes more accurate; however, in practice for circles of small radius, the discretization of LAT data sets a limit on the number of independent chords that can be defined. Additionally, if the radius of the circle is chosen too large, the plane wave approximation no longer holds and Eq. 1 is no longer exact. This geometric relationship gives

us a way to estimate the true conduction speed from measurements of conduction speed along chords at orientations not aligned directly with the true propagation direction.

A more accurate determination of the true conduction speed can be achieved by incorporating estimates of the wave's conduction speed calculated from measurements of conduction speed along several chords not oriented along the propagation direction. This can be done with a simple mean over several estimates calculated from Eq. 1 as

$$S_{\mathrm{CV}} = \frac{1}{N}\sum_{n=1}^{N}\frac{S(\theta_n)}{\cos\left(\theta_{PD}-\theta_{\mathrm{n}}\right)}, \quad (2)$$

where n goes over all N chords lying within the range $\theta_{PD} - \Delta\theta/2$ to $\theta_{PD} + \Delta\theta/2$.

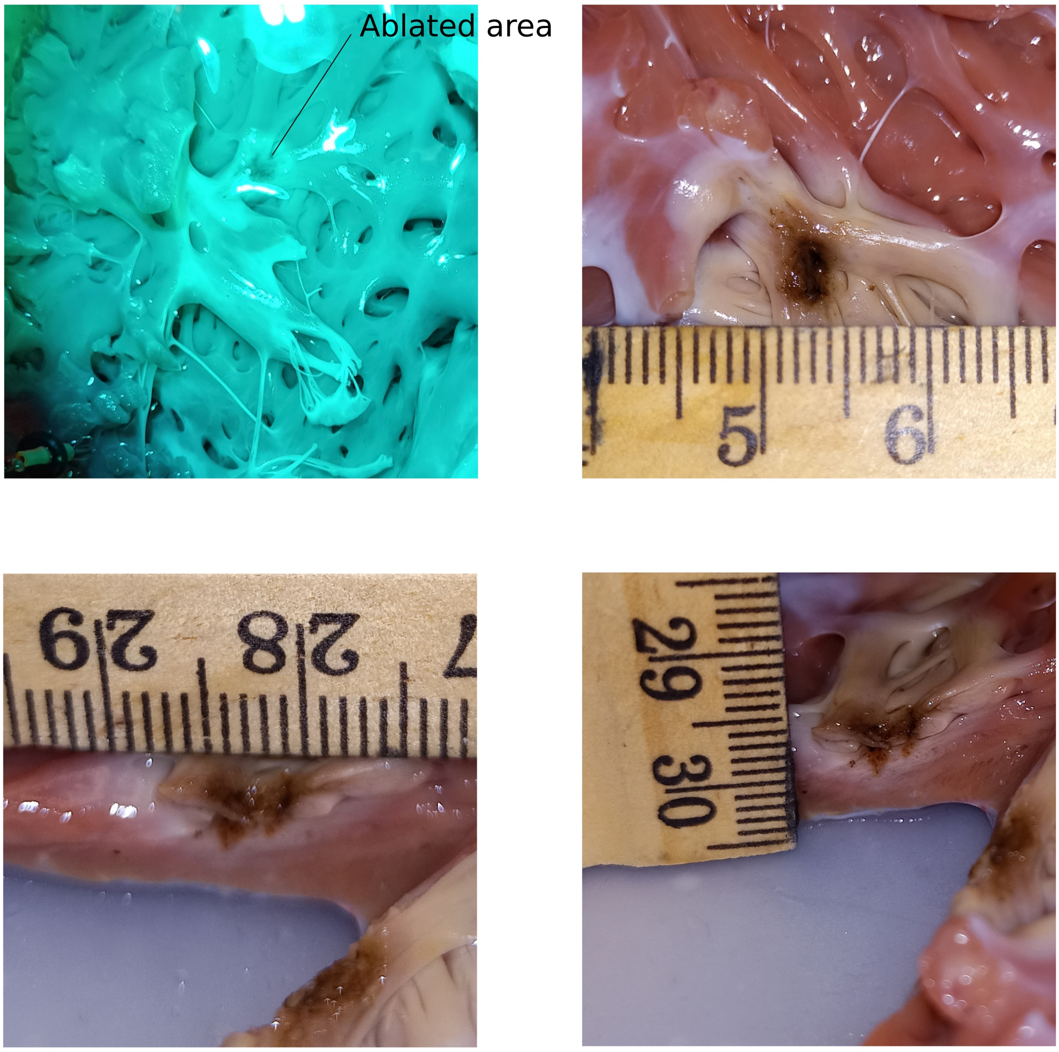

Supplemental Figure 2.
